# A molecular catalyst for water oxidation that binds to metal oxide surfaces

**DOI:** 10.1038/ncomms7469

**Published:** 2015-03-11

**Authors:** Stafford W. Sheehan, Julianne M. Thomsen, Ulrich Hintermair, Robert H. Crabtree, Gary W. Brudvig, Charles A. Schmuttenmaer

**Affiliations:** 1 Department of Chemistry, Yale University, 225 Prospect Street, PO Box 208107, New Haven, Connecticut 06520-8107, USA; 2 Centre for Sustainable Chemical Technologies, University of Bath, Claverton Down BA2 7AY, UK

## Abstract

Molecular catalysts are known for their high activity and tunability, but their solubility and limited stability often restrict their use in practical applications. Here we describe how a molecular iridium catalyst for water oxidation directly and robustly binds to oxide surfaces without the need for any external stimulus or additional linking groups. On conductive electrode surfaces, this heterogenized molecular catalyst oxidizes water with low overpotential, high turnover frequency and minimal degradation. Spectroscopic and electrochemical studies show that it does not decompose into iridium oxide, thus preserving its molecular identity, and that it is capable of sustaining high activity towards water oxidation with stability comparable to state-of-the-art bulk metal oxide catalysts.

## Abstract (web summary)

Molecular catalysts can exhibit high activity, but their practical application may be limited by solubility and stability issues. Here, the authors report stable, direct binding of an iridium molecular catalyst to conductive oxide surfaces and demonstrate its activity for electrochemical water oxidation.

## Graphical abstract

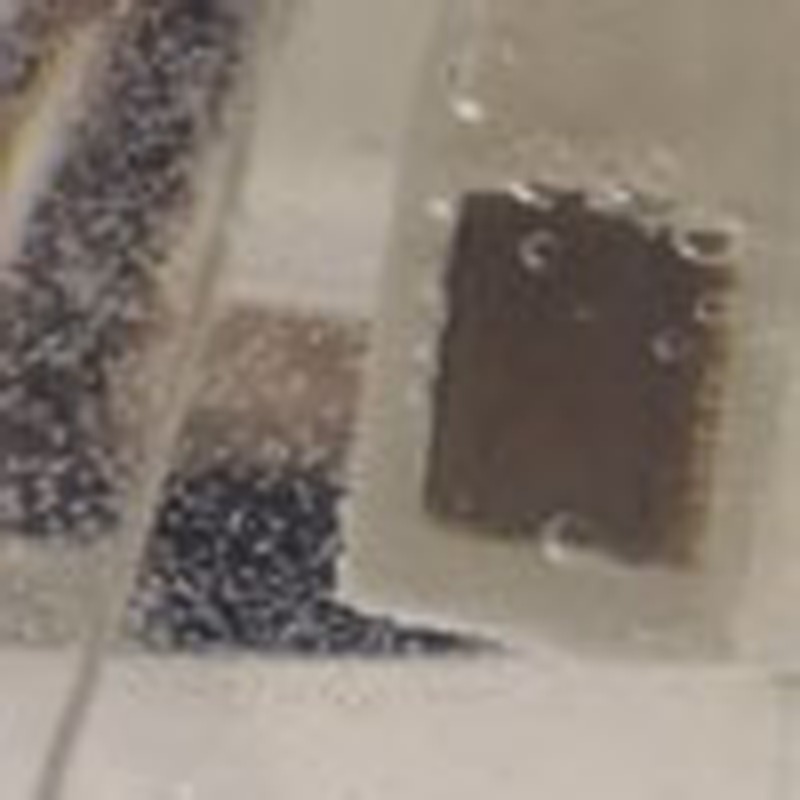

Economic and environmental concerns raised by the extensive use of fossil fuels have made alternative energy sources more attractive^1^. Renewable sources are particularly promising owing to their environmental sustainability and potential for widespread availability. However, there are a number of problems that need to be addressed before renewable energy can be used on a global scale^2^. Chief among these is the lack of reliable methods of concentrating and storing energy on a large scale, since numerous renewable energy sources, including solar and wind, are intermittent and diffuse. Towards this end, the generation of renewable fuels, in which electricity generated by renewable means is stored in the chemical bonds of a suitable fuel, has become a critically important area of research^3^. In one scheme for the formation of renewable fuels that mimics photosynthesis in plants, protons and electrons are extracted from water by electrochemical oxidation to be used for fuel formation, liberating O_2_ as a byproduct^4^. The efficient generation of such renewable fuels, therefore, necessitates the development of efficient, fast and stable water-oxidation catalysts (WOCs).

Of the extensive library of available WOCs, molecular species show promise because of their high activity and tunability, as well as their ability to be integrated into sophisticated molecular assemblies^5^,^6^,^7^,^8^,^9^,^10^,^11^,^12^. Their major drawback is their limited stability, with the best homogeneous systems providing turnover numbers in the thousands^13^,^14^,^15^ to tens of thousands^16^. This problem is particularly pronounced for electrode-driven WOCs, which often decompose to less active materials under moderate applied potentials^17^,^18^. Building on the success of different heterogenization strategies for homogeneous catalysts in organometallic, inorganic and surface chemistry^19^,^20^,^21^,^22^,^23^,^24^,^25^,^26^,^27^, immobilization of molecular WOCs on electrode surfaces has been sought to overcome this^28^,^29^,^30^. However, in the case of an electrode-driven WOC, the ligand anchoring the catalyst to the electrode surface must display a high degree of oxidative stability, which is not always the case^31^. Methods that alter the electrode surface, including deposition of coating layers of TiO_2_ after catalyst adhesion have been shown to assist in solving this issue^32^. However, to date, no system has fully succeeded in combining the high efficiency and tunability of a molecular catalyst that contains a single, well-defined catalytically active site (single-site)^33^ with the durability and stability of a bulk material in a heterogeneous electrocatalyst for water oxidation.

In a recent report^34^, we identified highly active homogeneous WOCs that are formed by the oxidative removal of Cp* (pentamethylcyclopentadienyl, C_5_Me_5_^−^), an organic placeholder ligand, from well-studied Cp*Ir based precursors^35^,^36^. The compounds that form from these precatalysts all possess a single bidentate chelate ligand^37^ per iridium that is oxidatively stable and prevents the formation of IrO_*x*_-based films^38^ or nanoparticles^39^ under oxidative conditions. In contrast, Cp*Ir precursors lacking a stable bidentate ligand anodically deposit amorphous IrO_*x*_ on electrodes to give a heterogeneous WOC referred to as ‘blue layer’ (BL)^38^. This BL, hydrated IrO_*x*_ nanoclusters^40^, and the homogeneous WOCs formed by the oxidative activation of our organometallic iridium precursors^41^,^42^ all display a characteristic deep blue colour in their oxidized form, owing to an absorption feature near 600 nm. This has caused confusion about the identity of the active species in these systems^34^,^39^; thus, in the following we refer only to the activated form of the molecular iridium catalyst as the hom-WOC.

In this study, we report the heterogenization of the hom-WOC to form a surface-bound, ligand-modified iridium electrocatalyst for water oxidation in acidic solutions. On self-adhering to the surface of a metal oxide at room temperature, a molecular monolayer of the catalyst is formed, which possesses higher activity than the bulk material analogue, IrO_*x*_. We show that this heterogenized molecular catalyst remains bound to the surface after extended use, eliminating the need for any linking moieties, while retaining its molecular identity and ligand-based tunability.

## Results

### Catalyst preparation and heterogenization

**Figure 1:**
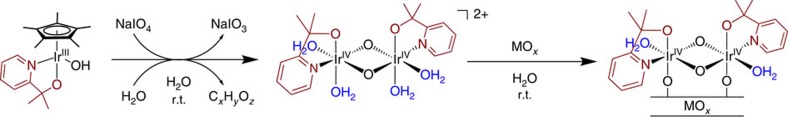
Formation of the hom-WOC and proposed molecular structure for the adsorbate. Oxidation of the [Cp*Ir(pyalc)OH] precursor (left) to form the [Ir(pyalc)(H_2_O)_2_(μ-O)]_2_^2+^ hom-WOC (middle) and heterogenization at room temperature (r.t.) to form the het-WOC (right) is shown.

In the pursuit of combining the high efficiency of the molecular hom-WOC with the stability of bulk metal oxides, we found that when an oxide material is immersed in an aqueous solution of the hom-WOC, the material rapidly and irreversibly chemisorbs some of the blue-coloured complex from the solution (we refer to this supported heterogeneous complex as the het-WOC). The dinuclear structures shown in Fig. 1 are based on the characterization data reported in this paper and on the prior work on the homogeneous analogue (hom-WOC)^34^,^41^. These structures are consistent with the available data, but are not intended to be definitive.

**Figure 2:**
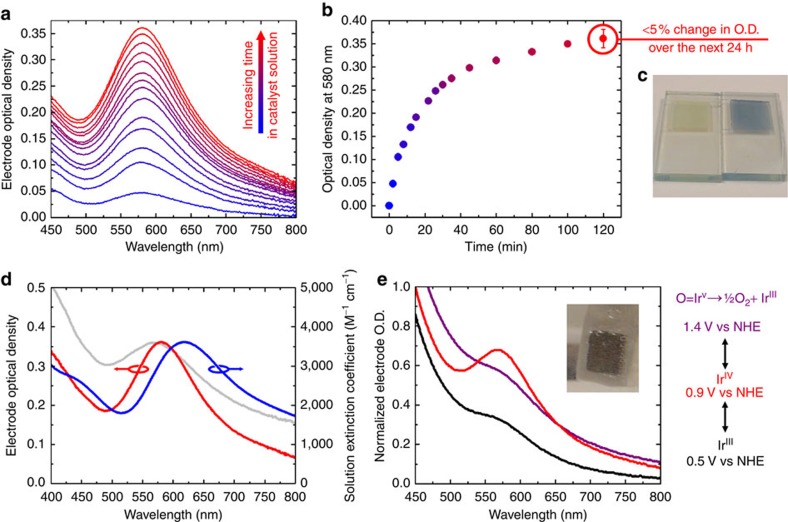
Spectroscopic characterization of the het-WOC on *nano*ITO electrodes. (**a**) Optical density (O.D.) spectra of an electrode measured after increasing the amounts of time immersed in hom-WOC solution at room temperature. Between each measurement, the electrode was washed thoroughly with deionized water. (**b**) Increase of O.D. at 580 nm for the electrode as a function of immersion time, error bar shows the largest measured deviation in optical density across 3 samples after further immersion for 24 h. (**c**) Photograph of an electrode before (left side of panel) and after (right side of panel) immersion in hom-WOC solution for 2 h. (**d**) Comparison of the spectra of the catalyst on the surface (red) to the catalyst in solution (blue) along with IrO_*x*_ electrodeposited on *nanoI*TO (grey). (**e**) Spectroelectrochemical response of the electrode showing reversible transitions between Ir^III^ (black) and Ir^IV^ (red) oxidation states, as well as under turnover conditions (purple). The absorption feature at 580 nm is characteristic of Ir^IV^, and the highly simplified legend on the right shows the potentials applied to reach these oxidation states. Inset is a photograph of the electrode under turnover conditions (1.4 V versus NHE, pH 2.6) corresponding to the spectrum in purple.

In contrast, the hom-WOC does not bind to noble metals that do not form a native oxide layer such as Au or Pt^41^. To further probe the electrochemical and spectroscopic properties of the het-WOC, we moved to high surface area transparent conductive electrodes consisting of mesoporous films of tin-doped indium oxide nanoparticles (*nano*ITO)^43^ on fluorine-doped tin oxide (FTO)-coated glass slides. A solution of the hom-WOC bearing a 2-(2′pyridyl)-2-propanolate (pyalc) bidentate ligand, previously characterized as [Ir(pyalc)(H_2_O)_2_(*μ*-O)]_2_^2+^, was prepared from [Cp*Ir(pyalc)OH] and NaIO_4_ using established methods^34^. On immersion, catalyst binding to the *nano*ITO surface is rapid, self-limiting and does not require any external driving force such as photons or an applied potential (Fig. 2a). Control experiments show that the removal of the organometallic placeholder ligand from the precursor is required for surface binding to occur in all the cases (Supplementary Figs 1–4). Formation of a molecular monolayer is complete in 2 h at room temperature, with negligible absorption changes being observed at later times (Fig. 2b). Even after thorough rinsing with deionized water, the catalyst is not washed off the surface. Transmission electron microscope (TEM) and scanning electron microscope (SEM) images of the electrode indicate that no nanoparticulate deposits are formed, and energy-dispersive X-ray spectroscopy (EDX) measurements confirm the presence of iridium on the electrode surface without any traces of iodine or sodium from the NaIO_4_ used to produce the hom-WOC in solution (Supplementary Figs 5–8).

The hom-WOC absorbance peak at 608 nm in solution blue shifts to 580 nm in the het-WOC formed on binding to *nano*ITO (Fig. 2d), both being distinct from electrodeposited IrO_*x*_^38^. The shift that occurs during catalyst heterogenization is similar to that previously observed for the reversible deprotonation of bound water ligands^34^. Along with the fact that the catalyst remains bound to the surface after repeated washing, this suggests chemical binding rather than mere physisorption. Both the pH and the concentration of iodate in the hom-WOC solution have pronounced effects on the rate of binding (Supplementary Fig. 9). As the pH of the solution is decreased, the rate of catalyst binding increases. Similarly, when the pH of the solution is increased, the rate of catalyst binding decreases, demonstrating that the catalyst binds faster when retaining aqua ligands rather than more strongly coordinating hydroxo ligands, consistent with a water displacement mechanism. These sites are also rapidly exchanged with the anion present in solution, iodate, which can also act as a ligand^44^. Consistently, we found that an increase in iodate concentration inhibits surface binding; however, a low concentration of iodate is required for heterogenization. When Cp*Ir precatalyst activation to form the hom-WOC is performed electrochemically^41^, surface binding does not occur unless iodate is present in the solution. Electron paramagnetic resonance spectroscopy of the het-WOC showed no signals that would be expected for monomeric Ir^IV^ species on the surface^45^, while X-ray photoelectron spectroscopy (XPS) proves that iridium is indeed present in the Ir^IV^ state (Supplementary Fig. 10)^46^,^47^,^48^. This is also the case for the hom-WOC in solution, indicating that the catalyst is still in dimer form when bound to the surface.

The absorbance peak at 580 nm is still evident after the electrode is immersed in aqueous solution in a spectroelectrochemical cell (Fig. 2e, Supplementary Figs 11–13). In this experiment, the catalyst-coated *nano*ITO working electrode forms a circuit with an Ag/AgCl reference electrode and a platinum counter electrode. We then vary the potential applied to the working electrode to induce reversible changes in the oxidation state of iridium in the het-WOC while collecting ultraviolet–visible spectra. Importantly, the catalyst remains bound to the electrode not only in its native Ir^IV^ state, but also in its reduced oxidation state, presumably Ir^III^, as well as the catalytically active state, presumably Ir^V^, from which oxygen evolution is observed^49^,^50^.

The stability and versatility of the het-WOC is shown by its irreversible adhesion when exposed both to acidic and basic aqueous solutions (pH values ranging from 1–12) and to numerous organic solvents, including CH_2_Cl_2_ and MeCN. Only repeated washing under highly alkaline conditions (pH>13) was found to remove the het-WOC from *nano*ITO, resulting in a clean electrode surface. The catalyst also adheres rapidly to different nanostructured metal oxides that are commonly used as photoanodes for light-driven water oxidation, including TiO_2_ and WO_3_ (Supplementary Fig. 14)^51^,^52^.

### Water-oxidation performance and stability

**Figure 3:**
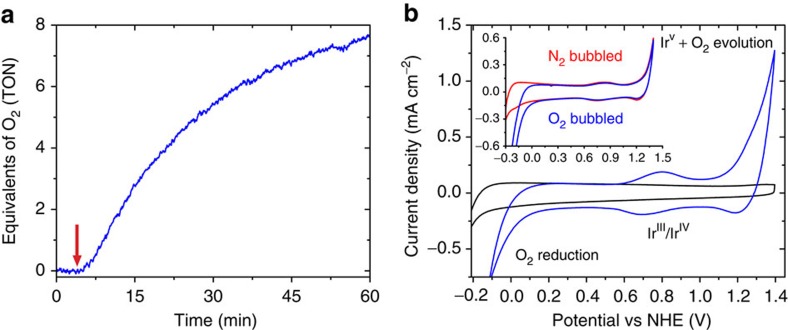
Water oxidation using the het-WOC. (**a**) Water oxidation using NaIO_4_ as a sacrificial oxidant with the catalyst bound to *nano*ITO; the red arrow corresponds to injection of NaIO_4_ solution, initiating catalysis as quantified by the turnover number (TON) of O_2_ per iridium atom on the mesoporous surface. (**b**) CVs of a catalyst-loaded *nano*ITO electrode (blue) compared with a similar *nano*ITO electrode without catalyst (black) in an oxygen-saturated solution of 0.1 M KNO_3_ in water at pH 2.6, taken with a 10 mV s^−1^ scan rate. Inset shows the effect of saturating O_2_ or N_2_ gas in solution when using thinner electrodes, highlighting the catalytic wave for oxygen reduction at 0 V versus NHE.

The het-WOC maintains its activity for oxygen evolution with chemical oxidants (Fig. 3a, Supplementary Fig. 15) compared with that previously observed^34^ for the hom-WOC in solution. Most importantly, however, the het-WOC shows exceptional activity when driven electrochemically. Cyclic voltammograms (CVs) in an oxygen-saturated solution of 0.1 M KNO_3_ in water at pH 2.6 (Fig. 3b, Supplementary Fig. 16) show reversible Ir^III^/Ir^IV^ charging features with E_1/2_=0.75 V versus the normal hydrogen electrode (NHE), as well as reversible water oxidation/oxygen reduction similar to traditional iridium oxides, but lacking a redox feature that has been assigned to the oxidation of Ir^IV^ to Ir^V^ (ref. 53). The onset of the water-oxidation catalytic wave occurs at a distinctively lower potential than the Ir^IV^/Ir^V^ redox couple in IrO_*x*_ samples prepared by different means^54^ and, thereby, obscures the Ir^IV^/Ir^V^ charging feature. Drawing a parallel to previously suggested mechanisms for Ir-catalysed water oxidation^36^,^49^,^50^, we postulate that this is a direct result of this catalyst’s highly active Ir^V^ state, which along with the high electroactivity of the molecular iridium compound on the surface, allows for water oxidation at low overpotentials. Specifically, integration of the Ir^III^/Ir^IV^ wave and comparison with the total iridium loading derived from ultraviolet–visible measurements demonstrates that >90% of iridium on the electrode is electroactive, as is expected for a molecular monolayer^55^.

Previously, we reported that the organometallic precursor complexes used to form the hom-WOCs by oxidative activation do not show any activity for electrode-driven water oxidation^41^. We also find that they do not self-adhere to oxide surfaces. Comparing both the activated forms, the kinetics of water oxidation appear to be different between the hom-WOC and the het-WOC: the H/D kinetic isotope effect (KIE) of 2.01 for the hom-WOC differs significantly from the KIE of 1.0 found for the het-WOC, when run at potentials below the appearance of mass-transport related effects at the electrode surface (Supplementary Fig. 17). This suggests that different rate-limiting steps are applied to each. The KIE of unity for the het-WOC may indicate that the rate-determining step is electron transfer from the Ir centres in the catalyst to the metal oxide scaffold, rather than any step involving water. KIEs that are close to 1 for similar reasons have been seen for iridium oxide colloids and related materials^36^,^56^.

**Figure 4:**
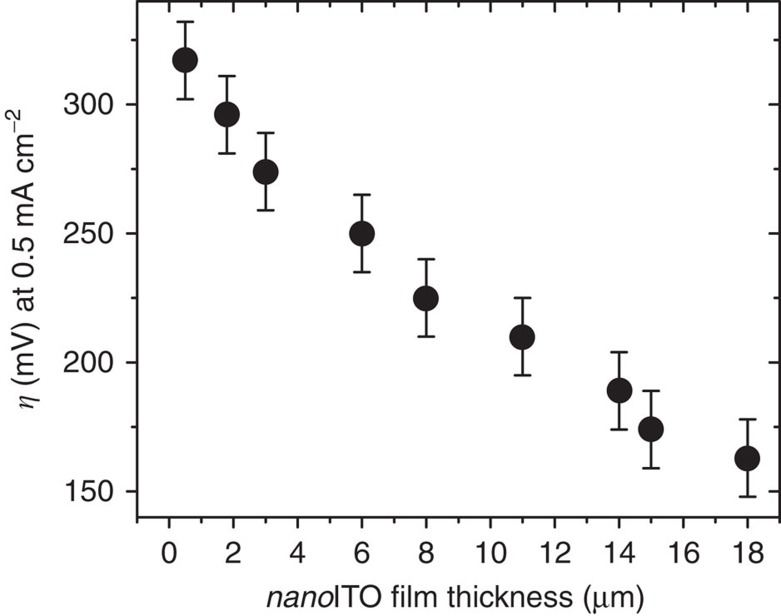
Electrode overpotential as a function of nanoporous film thickness. The film thickness is directly proportional to catalyst loading. As we increase the catalyst loading on the electrode, the overpotential required to reach 0.5 mA cm^−2^ decreases. Error bars reported represent the largest s.d. (sample size of five electrodes) across the nine film thicknesses measured. Data were gathered using 5-min dwell times to allow the electrodes to adequately stabilize.

In comparison with bulk IrO_*x*_ species, one advantage of using single-site surface-bound molecular catalysts for water oxidation is accurate control of electrode overpotential by tuning the scaffold surface area, thereby changing catalyst loading. By increasing the nanoporous film’s thickness, the overpotential of the electrode at specific current densities can be decreased (Fig. 4). For example, typical 3-μm-thick *nano*ITO films require an overpotential of 275 mV to attain a catalytic current of 0.5 mA cm^−2^, whereas 18-μm-thick films require <160 mV. Although limited tunability of the number of active sites in bulk or nanostructured WOCs prevents direct comparison, we are not aware of any lower overpotential values reported in the literature for this current density. Standardized benchmarking experiments comparing the het-WOC to IrO_*x*_ show that the het-WOC possesses a lower overpotential for water oxidation in all cases (Supplementary Fig. 18)^57^. However, any comparison between single-site molecular species and bulk heterogeneous catalysts is complicated because of the difficulty of determining the turnover rates per metal atom in bulk materials needed to accurately gauge the relative activity on a fair basis.

**Figure 5:**
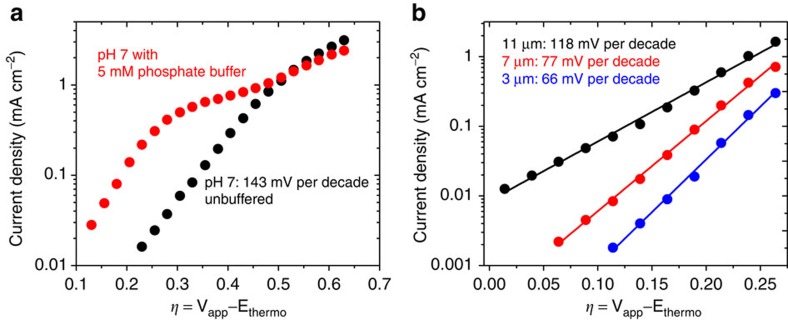
Tafel plots for the het-WOC on *nanoI*TO. (**a**) Tafel plots at pH 7 showing the effect of adding a phosphate buffer to the 0.1 M KNO_3_ solution. (**b**) Tafel plots at pH 2.6 without any added buffer. A decrease in Tafel slope (Δη /Δlog(*i*)) with decreasing film thickness corresponds to a decrease in diffusion-related pH effects.

To further investigate the het-WOC mechanistically, Tafel plots of catalytic currents were made over a range of pH and buffer conditions (Fig. 5a) and with electrodes of varying thickness of the porous *nano*ITO film to increase catalyst loading by increasing the electrode surface area (Fig. 5b). Limitations on proton diffusion through the nanoporous films on electrodes^58^,^59^ cause a decrease in measured activity due to the low buffering capacity of KNO_3_ at pH 7. As thicker *nano*ITO films are used, the pH gradient formed through the film decreases electrode performance over time regardless of the catalyst’s stability, because the locally generated highly acidic conditions etch the ITO support^60^. The use of a buffer may inhibit this effect, and in the presence of phosphate the het-WOC behaves in a manner identical to IrO_*x*_ materials^61^. Along with similarities in their pH dependence (Supplementary Fig. 19), CVs and spectroelectrochemical measurements, these results support the hypothesis that the active catalytic sites in iridium oxides such as BL are mechanistically similar to those in the het-WOC. At a pH of 2.6, where pH is less sensitive to proton production from water oxidation, Tafel slopes increase as *nanoI*TO film thickness is increased since the protons generated from water oxidation must diffuse through a thicker film. Remarkably, however, as seen in Fig. 5b, 11-μm-thick samples have high enough catalyst loading to allow sustained current densities with an onset of linearity in the Tafel plot beginning at overpotentials as low as 14 mV (where the current density is 11 μA cm^−2^). This is consistent with the onset of water oxidation (*E*_cat_) for this catalyst being at nearly the thermodynamic potential, though the current is below the threshold of 0.5 mA cm^−2^ required for practical use.

**Figure 6:**
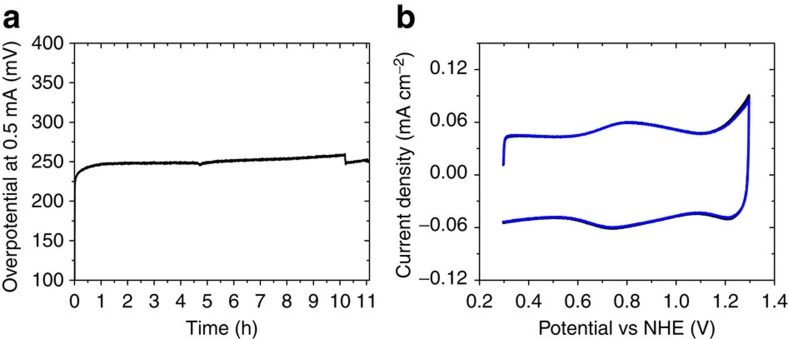
Stability for electrochemical water oxidation. (**a**) Chronopotentiometry showing stability of the het-WOC during sustained water oxidation for over 11 h (pH 2.6). Small increases and decreases correspond to oxygen bubble build-up and release on the surface, which was minimized by rapidly stirring the solution. (**b**) CVs (pH 2.6, 10 mV s^−1^ scan rate) after 1 h of electrolysis (black) and after >12 h of electrolysis (blue) using electrodes thin enough to mitigate local pH effects, showing full preservation of the electrode characteristics after sustained use.

The het-WOC also shows excellent stability, and is capable of sustaining water oxidation for many hours at a 250 mV overpotential without degradation (Fig. 6a, Supplementary Figs 20–22), reaching turnover numbers in excess of 10^6^ O_2_ evolved per iridium atom over multiple trials, as calculated by measuring the current passed through the electrode assuming a four-electron oxidative process. CVs of the electrode during and after these stability tests confirm that there is minimal loss in catalyst on the electrode surface (Fig. 6b). Moving to higher applied potentials (+520 mV relative to thermodynamic) we measure a turnover frequency of 7.9 s^−1^ O_2_ molecules evolved per electroactive iridium atom, which is one of the highest values reported to date. In addition, a 99% Faradaic yield is measured for O_2_ evolution over 2  h using a phase fluorometric oxygen sensor (Supplementary Figs 23 and 24). To compare this with benchmark iridium oxide nanomaterials reported in the literature, films of 2 nm IrO_*x*_ clusters with comparable electroactivity require an overpotential of 680 mV to achieve a turnover frequency of 6.0 s^−1^, while larger 60–100 nm IrO_2_ nanoparticles having 16% electroactivity require 580 mV to achieve a rate of 6.6 s^−1^ O_2_ molecules evolved per electroactive iridium atom^53^,^62^. The observed high performance and atomic efficiency further distinguishes this molecular iridium WOC from traditional bulk iridium oxides.

**Figure 7:**
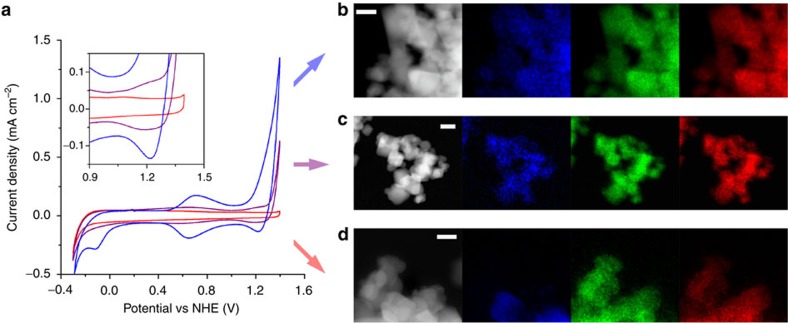
Comparison of iridium oxide catalysts on *nano*ITO films. (**a**) CVs taken with a 10 mV s^−1^ scan rate at pH 2.6 of a catalyst-coated *nano*ITO electrode as-prepared (blue), heated to 500 °C (purple) and heated to 700 °C (red). Inset is the 0.5–0.9 V versus NHE region expanded to more easily compare the features in the purple and red traces. (**b**) STEM-EDX maps of electrode material as-prepared: high-angle annular dark field image (grey), iridium detected shown in blue, indium in green and tin in red. (**c**) STEM-EDX map after heating to 500 °C showing iridium remains coated around the ITO nanoparticle scaffold after the pyalc ligand is burned off (X-ray photoelectron spectra shown in Supplementary Fig. 27). (**d**) Corresponding maps after heating to 700 °C, the iridium is now localized in specific regions indicating nanoparticle formation (scale bars, 20 nm).

To further probe the molecular nature of the het-WOC, we compare it with iridium oxide-based materials formed by heating an as-prepared electrode to 500 or 700 °C for 1 h (Fig. 7a). Scanning TEM analysis coupled with EDX mapping (STEM-EDX) displays the nanoscale coverage of iridium on ITO nanoparticles. As deposited, there is a highly conformal coating of iridium on each particle, consistent with a surface-bound molecular monolayer (Fig. 7b, Supplementary Fig. 25). The corresponding CV has a catalytic wave for water oxidation beginning at 1.1 V versus NHE at pH 2.6 (1.25 V versus RHE). Heating an electrode covered with the molecular catalyst at 500 °C in air burns off the pyalc ligand without affecting conformal coating of iridium oxide around each ITO nanoparticle (Fig. 7c, Supplementary Figs 26 and 27). This causes an anodic shift in the catalytic wave for water oxidation to 1.3 V versus NHE, revealing a feature at 1.1 V versus NHE typically assigned to the Ir^IV^/Ir^V^ redox couple^53^,^54^; in the unheated sample, the catalytic wave for water oxidation obscures this feature. Heating an electrode coated with the molecular catalyst to 700 °C in air results in the formation of crystalline rutile IrO_2_ clusters with ~20 nm diameter (Fig. 7d, Supplementary Fig. 28). In accordance with literature precedent^63^, these show even lower activity for water oxidation, in part because most of the iridium is no longer in contact with water, reducing the number of active surface sites.

### Molecular nature of the het-WOC

**Figure 8:**
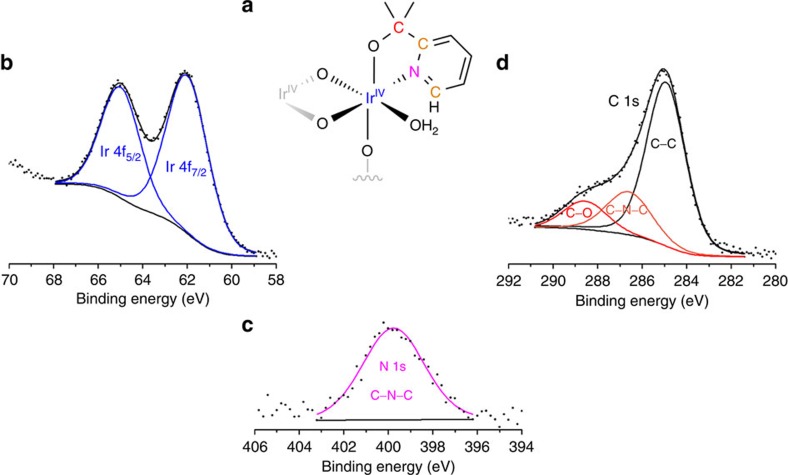
X-ray photoelectron spectra taken after 16 h of water-oxidation catalysis. Representative colour-coded schematic (**a**) showing elements present. Signals corresponding to the iridium (**b**), nitrogen (**c**) and carbon (**d**) show that both the metal and ligand are still intact after prolonged electrolysis. No changes are observed on the same film before and after electrolysis.

Aside from the monolayer distribution of active iridium, the oxidatively resistant bidentate pyalc ligand bound to iridium in the het-WOC represents another striking difference between single-site molecular catalysts such as this and traditional iridium oxide materials. Therefore, it is important to show that the ligand remains after extended periods of electrolysis, demonstrating that the het-WOC is stable. XPS, thus, serves to confirm the molecular structure and stability of the catalyst on the electrode, showing that the pyalc ligand is still present after ~16 h of electrolysis corresponding to >100,000 turnovers of O_2_ per iridium atom (Fig. 8, Supplementary Fig. 29, Supplementary Table 1). From these data, we can also conclude that the resting state for the catalyst on the electrode surface involves Ir^IV^ with a 1:1 ratio of pyalc to Ir as found previously for the hom-WOC in solution^34^.

Molecular catalysts are tunable by the variation of their ligands, and the het-WOC behaves in the same way. By changing the bidentate chelating ligand in the hom-WOC before deposition on the electrode surface, the properties of the het-WOC-functionalized electrode can be drastically changed. If, for example, [Cp*Ir(bpy)OH]BF_4_ bearing the 2,2’-bipyridine (bpy) ligand instead of pyalc is used as a precatalyst to form the active catalyst in solution^34^, a semitransparent yellow iridium compound (Supplementary Fig. 30) is deposited, having electrochemical properties that are very distinct from those of the pyalc-derived het-WOC (Supplementary Fig. 31), with much lower oxygen-evolution activity being observed (Supplementary Fig. 32). We find similar differences in activity in both electrochemically and chemically driven oxygen evolution for these two hom-WOCs in solution^41^. This shows that our heterogenization strategy preserves the ligand-based tunability of the hom-WOC.

## Discussion

These results provide a framework for assembling surface-bound molecular catalysts with a variety of direct-binding schemes^64^,^65^. The mild conditions of deposition are particularly promising with regards to nanostructured electrode materials that are difficult to functionalize by other means^66^. The higher activity and stability of the described materials over previously reported heterogenized molecular WOCs^67^,^68^ shows that direct surface binding is a valid approach to attaching WOCs to electrodes. The present system also outperforms heterogeneous IrO_*x*_ materials such as BL as a WOC, although they do share similar characteristics, and our further studies will determine how this molecular species mechanistically relates to the active sites in bulk iridium oxides^38^,^69^. The het-WOC requires minimal iridium to efficiently oxidize water relative to IrO_*x*_ catalysts, and although water-oxidation catalysis on a global scale as required for solar fuel production will likely require the use of catalysts made from more abundant elements, it is still advantageous to develop WOCs based on rare but highly active metals^70^. Nevertheless, this demonstration of a robust and highly efficient iridium-based molecular heterogeneous catalyst provides a new architecture for molecular heterogeneous catalysts and opens up this field to develop WOCs made from abundant materials using similar design principles.

## Methods

### General procedures

All the chemicals were purchased from major suppliers and used as received. Synthesis of the precatalyst [(*η*^5^-pentamethylcyclopentadienyl)Ir^III^(2-(2’pyridyl)-2-propanolate-κO,κN)OH] was performed using a published procedure^39^ and its activated form, the proposed [Ir(pyalc)(H_2_O)_2_(μ-O)]_2_^2+^ compound, was synthesized by the oxidation of the precatalyst with 100 equivalents of NaIO_4_ (Acros Organics, 99%) following previously published methods^34^.

### Electrode preparation

*Nano*ITO electrodes were prepared by spin coating (Headway PWM32 Spin Coater, Headway Research Inc.) a solution of ITO nanoparticles (Sigma-Aldrich, <50 nm particle size) suspended in a 5 M acetic acid/ethanol solution onto a 2.2 mm-thick FTO-coated glass slide (FTO, TEC 7, Hartford Glass Co. Inc.), followed by heating at 500 °C in air for 1 h, cooling to room temperature, then heating to 300 °C in a 3% H_2_/N_2_ atmosphere for 1 h and cooling back to room temperature. They were immersed in a catalyst solution formed by oxidizing 10 mM [Cp*Ir(pyalc)OH] in 30 ml deionized water with 100 equivalents of NaIO_4_. The electrodes were removed after 2 h, washed thoroughly with deionized water, and had acquired a visible blue colour. Side-by-side controls were used to measure the relative decrease in absorption of the catalyst solution, to approximately determine the amount of iridium that had adhered to the surface of the electrode. *Nano*ITO film thicknesses were measured using a profilometer (KLA Tencor Alphastep 200). Additional preparatory procedures for electrodes made from TiO_2_ and WO_3_, as well as the procedure for determining catalyst loading are detailed in the Supplementary Methods.

### Ultraviolet–visible spectroscopy

For optical studies including the data gathered in Fig. 2a,b,d, a Varian Cary 3 spectrophotometer with an integrating sphere attachment in absorption mode was used. A 6.45 cm^2^ geometric surface area of *nano*ITO on FTO-coated glass was used to completely cover the aperture of the integrating sphere. A background spectrum was taken before immersion of the substrate into the hom-WOC solution. Ultraviolet–visible spectra for IrO_*x*_ on *nano*ITO were taken by electrodepositing IrO_*x*_ from a solution containing [Cp*Ir(H_2_O)_3_]SO_4_ using the conditions outlined in ref. 38 with 50 deposition cycles at 50 mV s^−1^ scan rate. For the data gathered in Fig. 2e, an electrochemical cell as described previously was assembled in a 1 cm^2^ quartz ultraviolet–visible cuvette attached to an integrating sphere in a Shimadzu UV-2600 spectrophotometer. Standard electrolyte conditions of 0.1 M KNO_3_ adjusted to pH 2.6 were used. Catalyst-coated *nano*ITO electrodes that were 6.45 cm long and 0.7 cm wide were prepared by cutting an FTO-coated glass slide with a 7 μm thick catalyst-coated *nano*ITO film to the appropriate dimensions. Working electrodes were constructed by attaching a copper wire to an exposed FTO surface on one side of the *nano*ITO-coated FTO slide using conductive epoxy (Fast Setting Conductive Silver Epoxy, SPI). Six hours were allotted for the conductive epoxy to cure, then non-conductive water-resistant marine epoxy (White Marine Epoxy, Loctite, 24 h allotted to cure) was applied on top of the conductive epoxy to prevent electrical contact between the wire leads and electrolyte, so that the only conductive component exposed was the catalyst-coated *nano*ITO. This was placed into the quartz cuvette along with Pt mesh counter and Ag/AgCl reference (Bioanalytical Systems, Inc.) electrodes adjacent to the integrating sphere, connected to a potentiostat (WavenowXV, Pine), and chronoamperometric experiments with the potentials detailed in Fig. 2e were performed. The electrodes were given 3 min to stabilize at that potential, then a ultraviolet–visible spectrum was taken using the integrating sphere in absorption mode. A blank *nano*ITO-coated FTO slide without catalyst was used for a background scan.

### Chemical oxidation

Oxygen was detected with a Clark electrode using a custom-made zero-headspace 10 ml glass cell, water jacketed for constant temperature. A Teflon cap through which the Clark electrode membrane contacted deionized water (adjusted to pH 2.5 using nitric acid) also held a catalyst-coated *nano*ITO film on FTO-coated glass sample that was submerged in the cell. Catalyst-loaded samples were prepared similarly to those used for electrochemistry, except with a 1.6 cm^2^ geometric surface area. The Clark electrode was allowed to stabilize while stirring the deionized water solution for 1 h before injection with an oxidizing solution of freshly prepared NaIO_4_ in deionized water. Catalyst response and O_2_ generation occurred immediately, without any lag phase. A typical experiment, such as is shown in Fig. 3a, used 25 μl of 0.25 M NaIO_4_ in deionized water. The oxygen content in the cell was monitored until oxygen evolution ceased, which for a loading of around 50 nmol of iridium (corresponding to a *nano*ITO film 11-μm thick) took ~90 min. Data were collected while stirring the solution to ensure steady state oxygen readings.

### Electrochemical characterization

Electrolyte pHs were adjusted using 1 M HNO_3_ or 1 M KOH. Electrochemical data were taken using 0.1 M KNO_3_ in deionized water as an electrolyte, adjusted to pH 2.6 unless stated otherwise, with an Ag/AgCl reference electrode (Bioanalytical Systems Inc.) and Pt mesh counter electrode. Measurements were taken using a Princeton Applied Research Versastat 4–400 potentiostat in a standard three-electrode configuration. Vigorous stirring was required in unbuffered solutions during long-term experiments to prevent etching of the *nano*ITO electrode under acidic conditions. Long-term stability testing and oxygen detection using phase fluorometry were performed in a two-chamber electrochemical cell, with working and counter electrode chambers separated by a glass frit. For these experiments, such as those shown in Fig. 6, low catalyst loading was achieved with 300–500 nm thickness *nano*ITO films on a 6.45 cm^2^ substrate, thereby minimizing local pH effects due to the low buffer capacity of KNO_3_ over the pH range studied. SEM and TEM data were taken both before and after electrolysis to determine that there were no changes to sample morphology and CVs were taken to ensure the minimal loss of electroactive catalyst over the course of an experiment. Further details on electrochemical methods and additional controls are included in the Supporting Information (Supplementary Figs 33–35).

### Electron microscopy

SEM images and SEM-EDX data were taken on a Hitachi SU-70 Analytical Scanning Electron Microscope. Images of the samples were taken both before and after electrolysis. TEM images, EDX data, high-angle annular dark field images and STEM-EDX maps were taken using a FEI Tecnai Osiris TEM operating at 200 kV. Samples were prepared by scraping *nano*ITO off of an electrode into deionized water, then suspending them onto a silicon monoxide coated TEM grid (Ted Pella).

### X-ray photoelectron spectroscopy

X-ray photoelectron spectra were collected using an Al anode (*hν*=1486.6 eV) and a double-pass cylinder mirror analyzer (PHI 15- 255G). Geometric surface area 6.45 cm^2^ samples with a ~400 nm thick *nano*ITO film on the surface of FTO-coated glass were used for XPS studies. All experiments used a pass energy of 35.75 eV. Spectra were calibrated to an Au standard, and peak fits were performed using XPSPeak (version 4.1). Additional information regarding peak fitting and experimental details for Supplementary Figures can be found in the Supplementary Methods.

## Author contributions

S.W.S. developed the concept of heterogenizing via direct binding the molecular homogeneous WOCs that were discovered previously by U.H., R.H.C. and G.W.B. S.W.S., J.M.T. and U.H. designed the experiments, S.W.S. carried out the electrode preparation, and S.W.S. and J.M.T. performed the experiments. S.W.S. wrote the manuscript and supporting information with contributions from J.M.T. U.H., R.H.C., G.W.B. and C.A.S. revised and edited the manuscript before submission. U.H., R.H.C., G.W.B. and C.A.S. supervised the work.

## Additional information

**How to cite this article:** Sheehan, S. W. *et al*. A molecular catalyst for water oxidation that binds to metal oxide surfaces. *Nat. Commun*. 6:6469 doi: 10.1038/ncomms7469 (2015).

## Supplementary Material

Supplementary Information Supplementary Figures 1-35, Supplementary Table 1, Supplementary Methods and Supplementary References
