# Supplementary material for: A molecular catalyst for water oxidation that binds to metal oxide surfaces
Source: Nat Commun. 2015 Mar 11;6:6469. doi: 10.1038/ncomms7469 (PMC4382695; doi:10.1038/ncomms7469)
Supplement: Supplementary Information — Supplementary Figures 1-35, Supplementary Table 1, Supplementary Methods and Supplementary References [file ncomms7469-s1.pdf]

Supplementary Figures

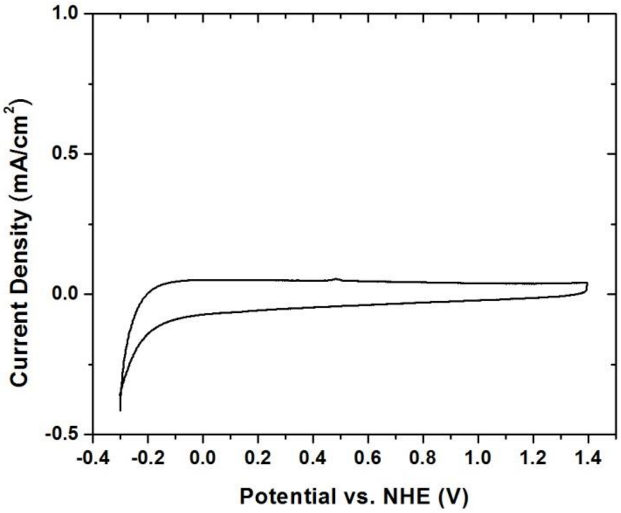

Supplementary Figure 1. CV under standard conditions (pH 2.6, 0.1 M KNO_3 in deionized water solution, 10 mV/s scan rate) of a *nano*ITO electrode after being immersed in a solution of 5 mM [Cp*Ir(pyalc)OH] for 12 hours, showing that before activation with $NaIO_4$ the pre-catalyst compound does not have the ability to bind to surfaces. Experiments repeated with higher concentrations of [Cp*Ir(pyalc)OH] (up to 20 mM) and longer times (up to 48 hours) gave the same result. The catalyic wave at -0.3 V vs NHE corresponds to reduction of the substrate.

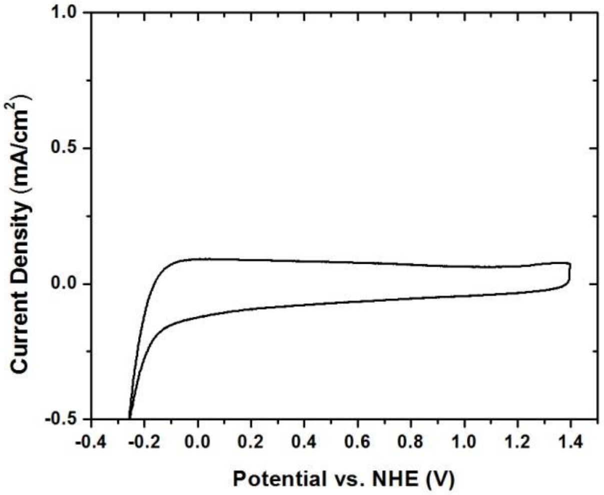

Supplementary Figure 2. CV in standard conditions of a *nano*ITO electrode that has been immersed in a 10 mM solution of the free pyalc ligand (2-(2'pyridyl)-2-propanol) for 12 hours.

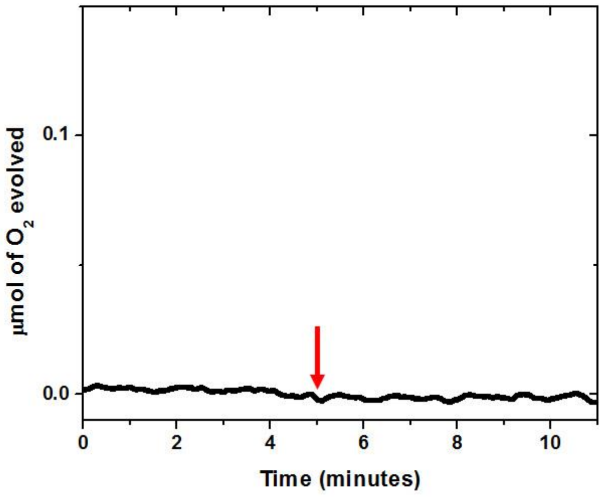

Supplementary Figure 3. Control experiment using an 11 μm thick film of *nano*ITO on FTO/glass sample that had been soaked in a solution of the precatalyst [Cp*Ir(pyalc)OH] in deionized water (10 mM). 25 μL of 0.25 M $NaIO_4$ in deionized water was used as an oxidant, and the red arrow corresponds to injection. We found no evidence that the precatalyst heterogenizes, prior to formation of the active species.

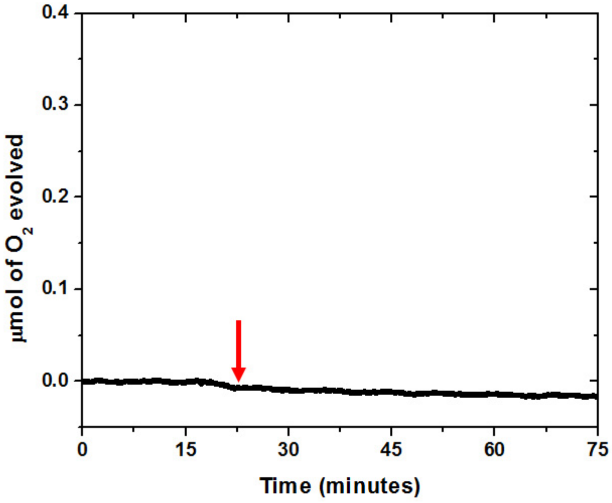

Supplementary Figure 4. Control experiment of a *nano*ITO on FTO/glass slide without catalyst for over one hour to show no catalytic activity from the substrate. The Clark electrode consumes oxygen, causing the baseline to slope downwards over long periods of time.

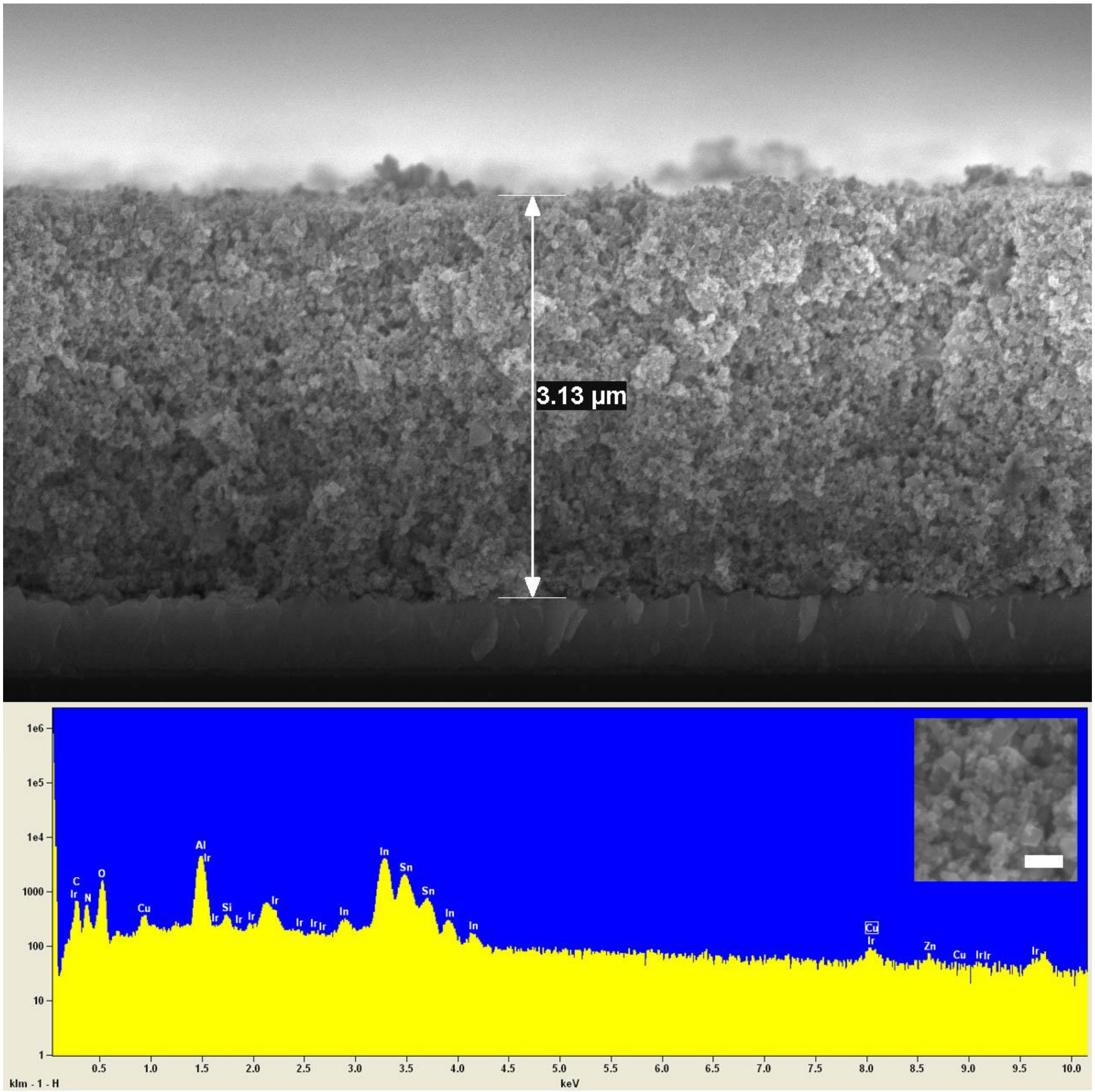

Supplementary Figure 5. Cross-sectional SEM image of the catalyst-loaded *nano*ITO working electrode (top), along with an SEM-EDX spectrum (bottom) showing presence of C, N, O, and Ir, in addition to In, Sn, Si, and O from the conductive oxides (ITO and FTO) on glass. Al, Cu, and Zn peaks arise from the sample holder. Inset is a higher resolution SEM image of the area scanned (scale bar: 100 nm), with only *nano*ITO particles of varied sizes visible, all having been coated with the Ir-based molecular WOC. No I or Na was detected.

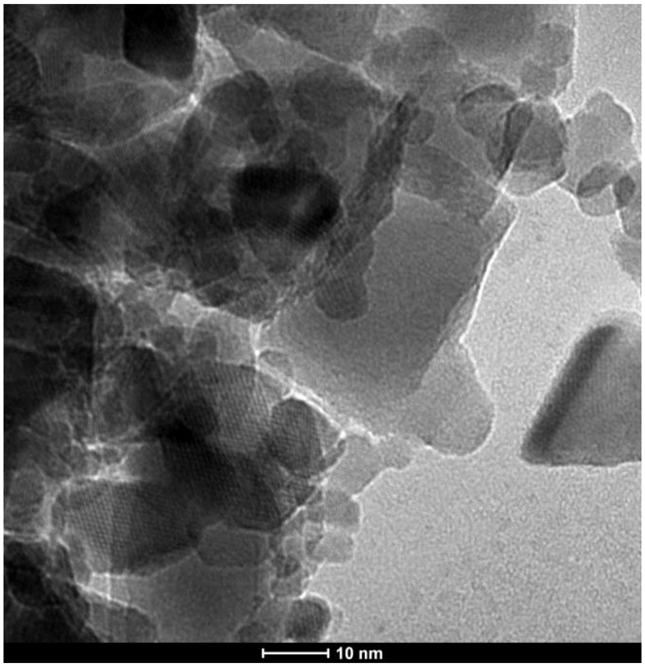

Supplementary Figure 6. TEM image of the catalyst-coated *nano*ITO.

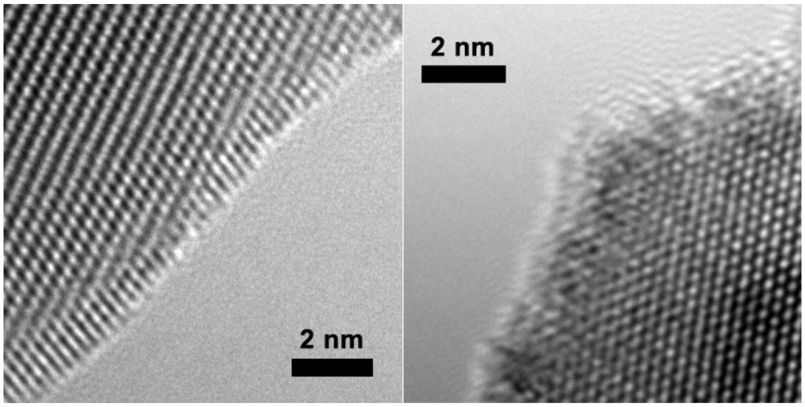

Supplementary Figure 7. HRTEM images of a bare *nano*ITO particle (left) compared to a *nano*ITO particle with **het-WOC** on the surface (right). In the image on the right, the catalyst is being slowly burned by the concentrated electron beam.

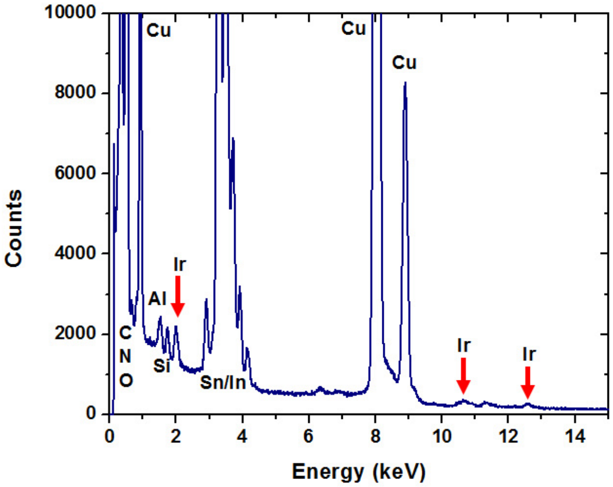

Supplementary Figure 8. TEM-EDX spectrum zoomed in with Ir peaks identified using red arrows (other peaks due to pyalc ligand, *nano*ITO substrate, SiO coated Cu TEM grid, and Al TEM sample holder).

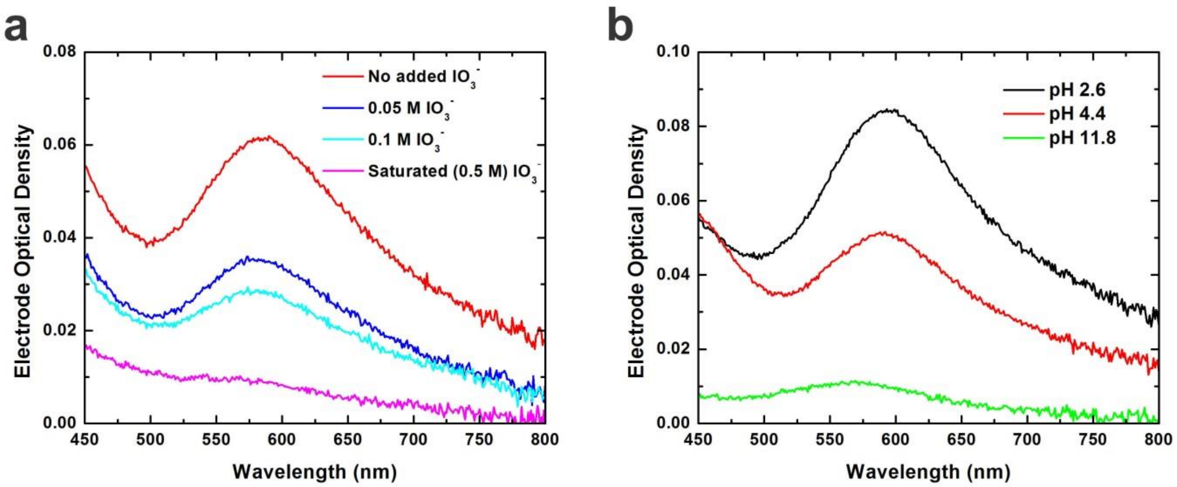

Supplementary Figure 9. Diffuse reflectance UV-Vis data taken on samples soaked in homogeneous catalyst solutions for 1 hour with (a) different concentrations of added $NaIO_3$, and (b) different pH conditions, adjusted using nitric acid.

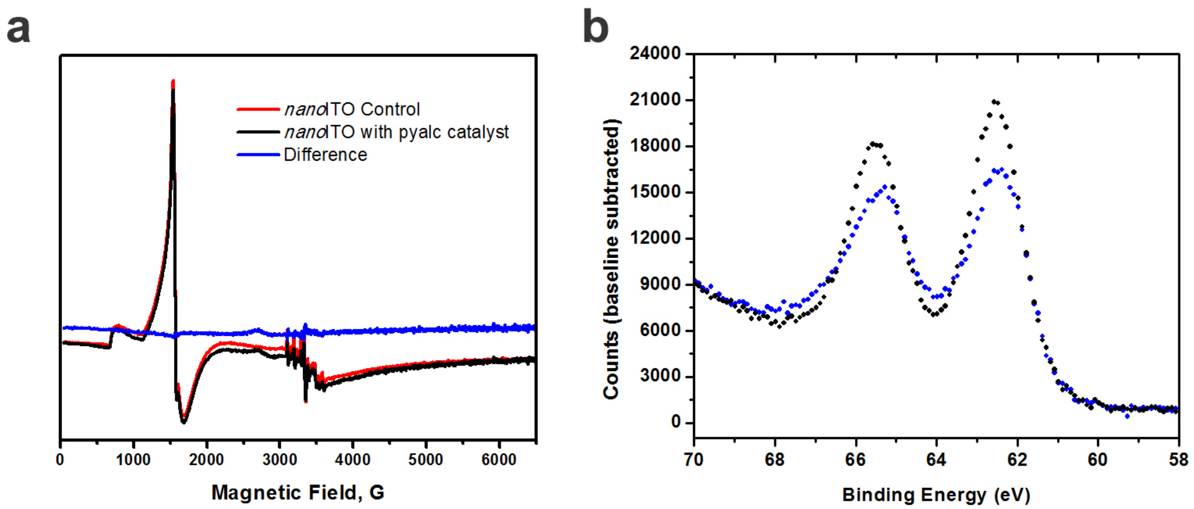

Supplementary Figure 10. (a) EPR spectrum at 8 K of the pyalc Ir^{IV}/Ir^{IV} dimer **het-WOC** on *nano*ITO. No Ir^{IV} signal is seen, in agreement with the solution phase data, suggesting that the Ir^{IV} centers remain bound in strongly coupled dimers. (b) XPS spectra of the Ir region of the **het-WOC** on *nano*ITO, showing that no significant peak shift occurs before (black dots) and after (blue dots) water electrolysis. This shows that Ir is present in the same oxidation state (IV) both as-prepared and post-electrolysis. Data was collected using a Thermo Scientific K-Alpha$^+$ XPS system; further experimental details are below in the Supplementary Methods section.

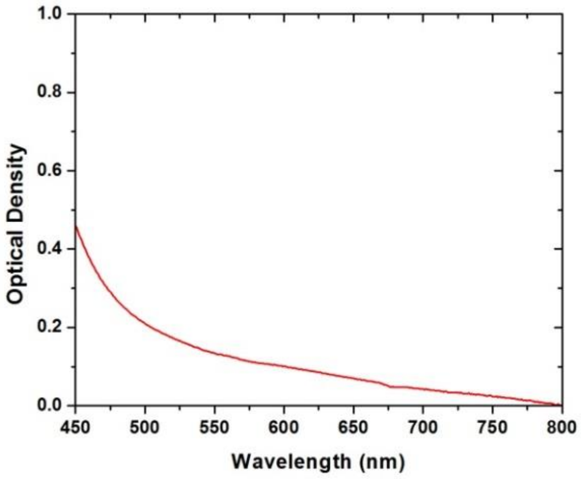

Supplementary Figure 11. Background UV-Vis spectrum of bare *nano*ITO on an FTO-coated glass slide taken using an integrating sphere in absorption mode.

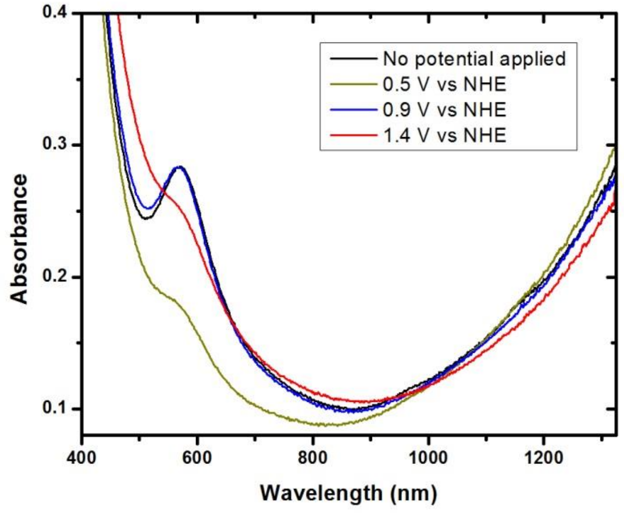

Supplementary Figure 12. Absorption spectra for the full range of spectroelectrochemical data shown in Figure 1e, without any normalization, and, in addition, a scan with no potential applied showing its similarity to that of a spectrum taken at a potential of 0.9 V vs NHE.

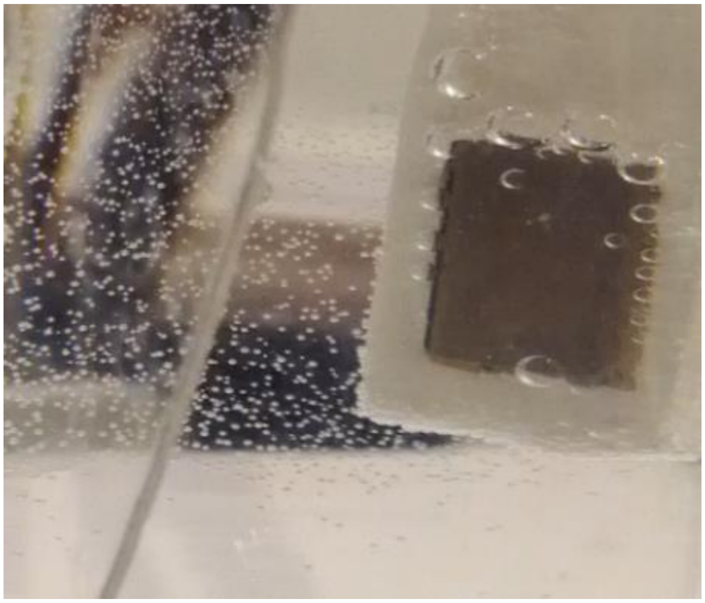

Supplementary Figure 13. Enlarged photograph of the electrode held at 1.4 V vs NHE, with a similar image shown in the inset of Figure 1e, with Pt wire evolving H_2 in the foreground. A distinct purple color is present only under catalytic conditions.

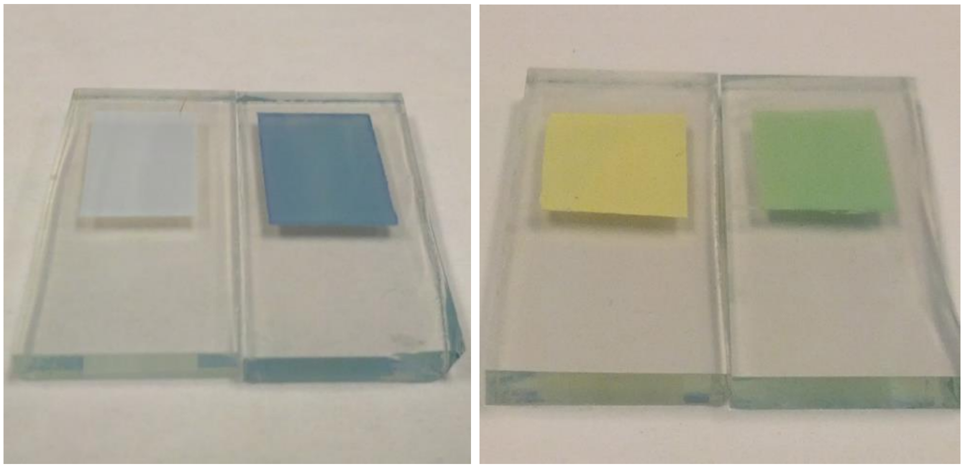

Supplementary Figure 14. Left image: TiO_2 electrodes without (left) and with (right) molecular iridium WOC bound to the surface. Right image: WO_3 electrodes without (left) and with (right) molecular iridium WOC bound to the surface.

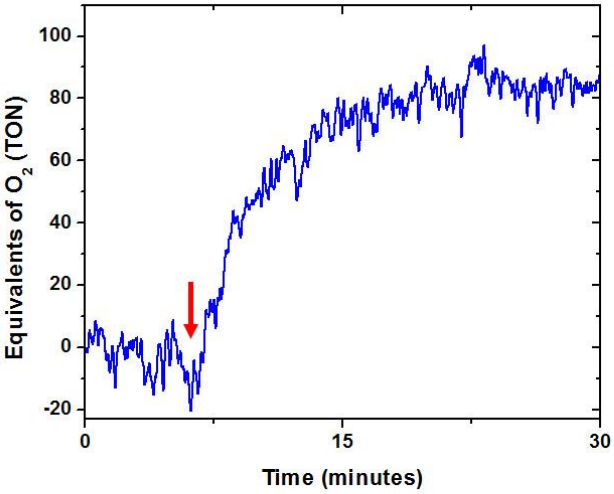

Supplementary Figure 15. Increased turnover of heterogenized catalyst using a chemical oxidant, achieved by decreasing *nano*ITO film thickness in the sample used thereby decreasing amount of Ir WOC relative to $NaIO_4$ oxidant (500 μL of 0.1 M $NaIO_4$ in deionized water, red arrow corresponds to injection). This sample contained ~1.6 nmol of iridium. This sample was prepared using a conductive epoxy and copper wire similarly to electrochemical samples, so that catalyst loading could be confirmed by taking a CV and integrating the Ir^{III}/Ir^{IV} peak which detected 1.55 nmol of electroactive Ir.

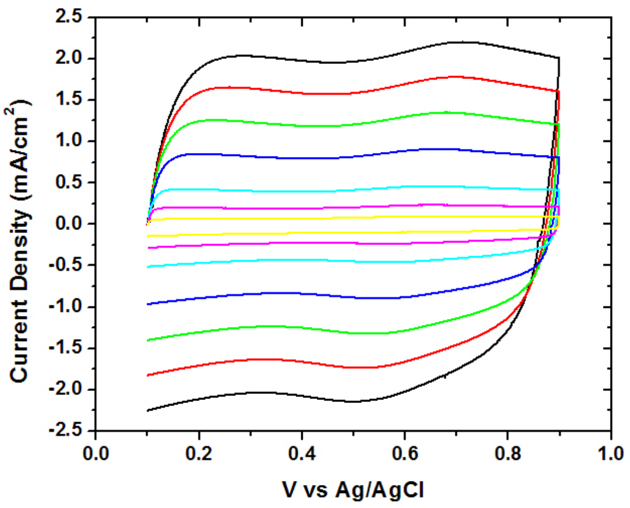

Supplementary Figure 16. To show that the surface-bound Ir WOC behaves electrochemically as a surface monolayer, the Ir^{III}/Ir^{IV} redox wave was investigated at different scan rates. Varied scan rate CVs for the heterogeneous Ir WOC on *nano*ITO. Traces correspond to scan rates as follows: yellow: 20 mV/s, pink: 50 mV/s, light blue: 100 mV/s, blue: 200 mV/s, green: 300 mV/s, red: 400 mV/s, black: 500 mV/s. Standard electrolyte conditions at pH 2.6 were used. . Integrals of the forward and reverse waves increased approximately linearly with scan rate.

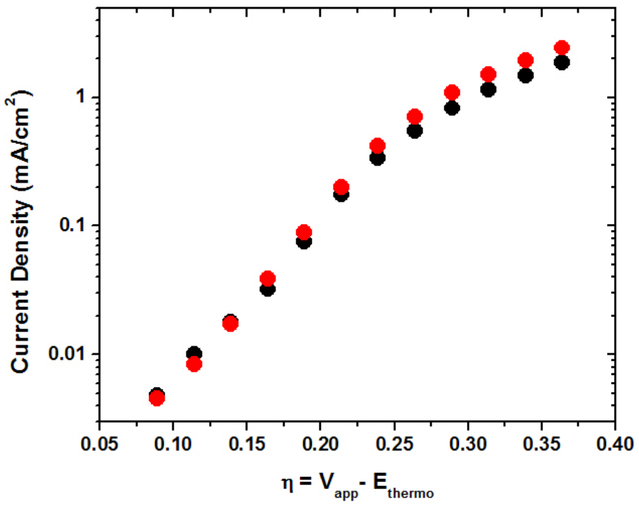

Supplementary Figure 17. Tafel plots taken in H_2O (red) and 99.9% D_2O (black) showing a KIE of approximately 1 below the onset of mass-transport associated limitations at $\eta > 0.25$ V.

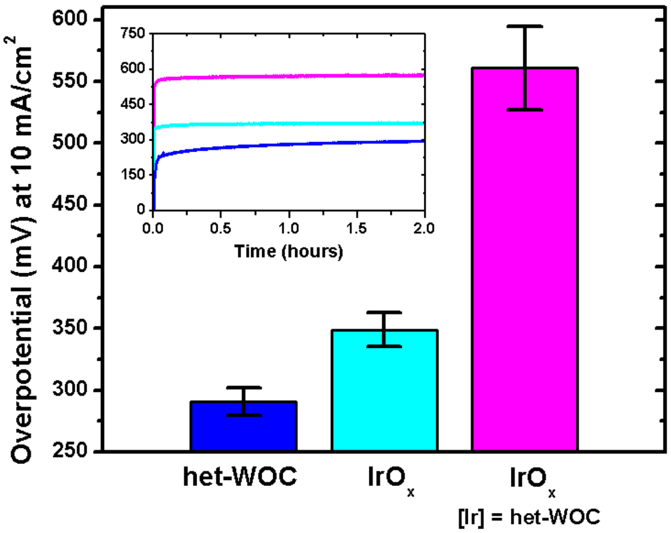

Supplementary Figure 18. Comparison of the overpotential at a constant current density of 10 mA/cm^2 for the **het-WOC** (shown in blue) as well as IrO_x at two different thicknesses. Cyan: IrO_x with an Ir loading of approximately 950 nmol/cm^2. Pink: IrO_x possessing the same loading of Ir as in the **het-WOC** samples tested, approximately 50 nmol/cm^2. Standard electrolyte conditions were used, with *nano*ITO-coated FTO/glass electrodes. Averages of 5 samples for each are shown, with standard deviation as the error bars. Inset: Raw chronopotentiometry data for each sample type at 10 mA/cm^2 current density.

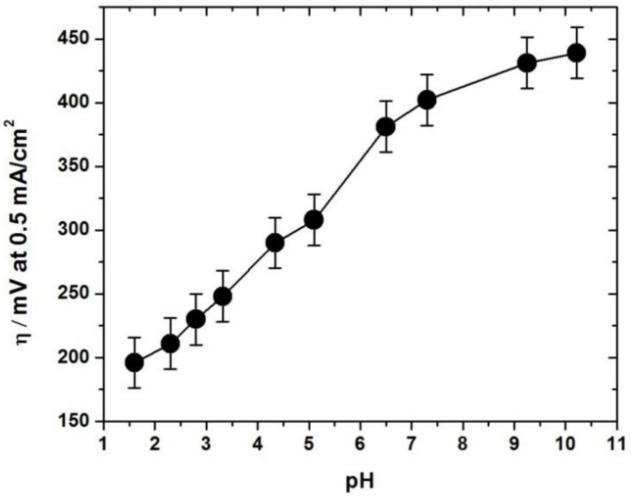

Supplementary Figure 19. pH dependence of the heterogenized Ir WOC.

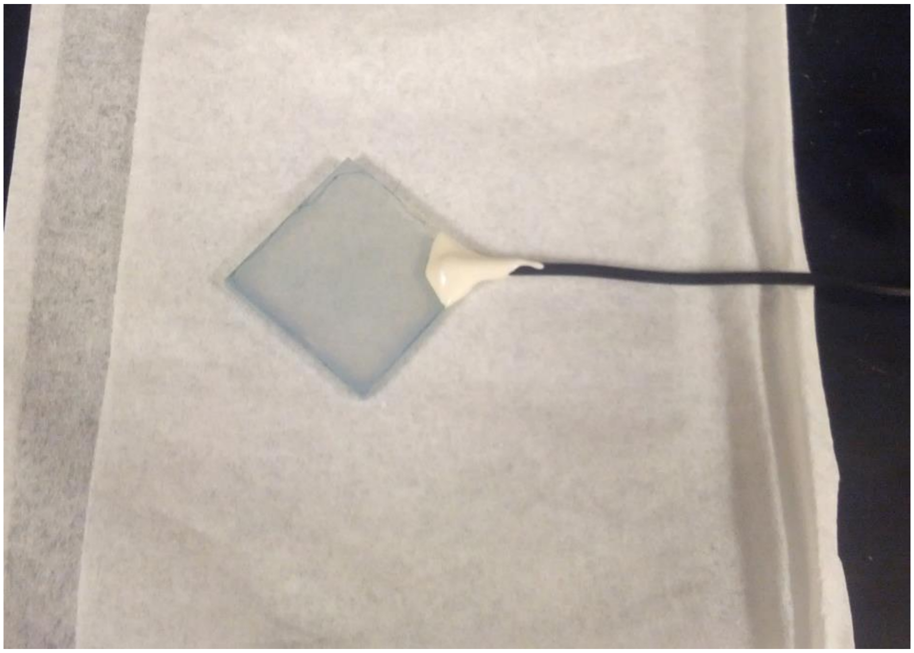

Supplementary Figure 20. Photograph of 6.45 cm^2 working electrode typically used for either oxygen detection and/or long-term stability testing.

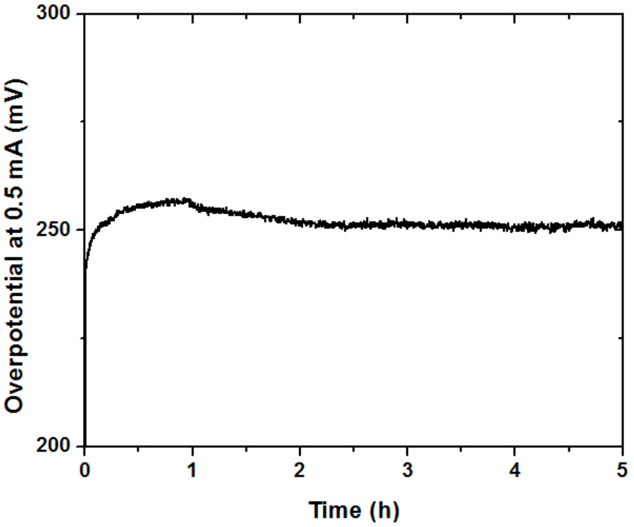

Supplementary Figure 21. Chronopotentiometric stability for an electrode prior to elemental and morphological analysis.

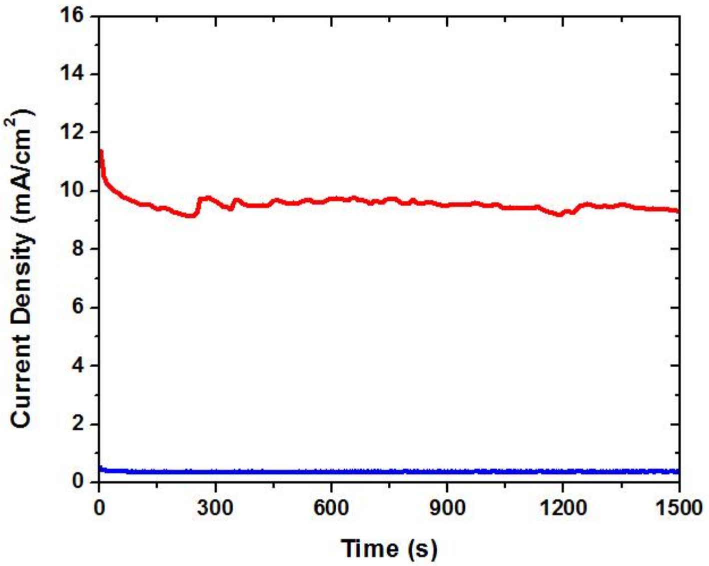

Supplementary Figure 22. Chronoamperometric stability at 2 V vs NHE of the catalyst on *nano*ITO on FTO/glass (red) vs a bare *nano*ITO control (blue) with catalyst loading approximately 2 nmol/cm^2, demonstrating that this catalyst can withstand high potentials for large-scale water splitting. Noise in the red trace is due to bubble formation and release, data were taken while rapidly stirring the electrolyte solution. Electrolyte conditions: 0.1 M KNO_3 at pH 2.5.

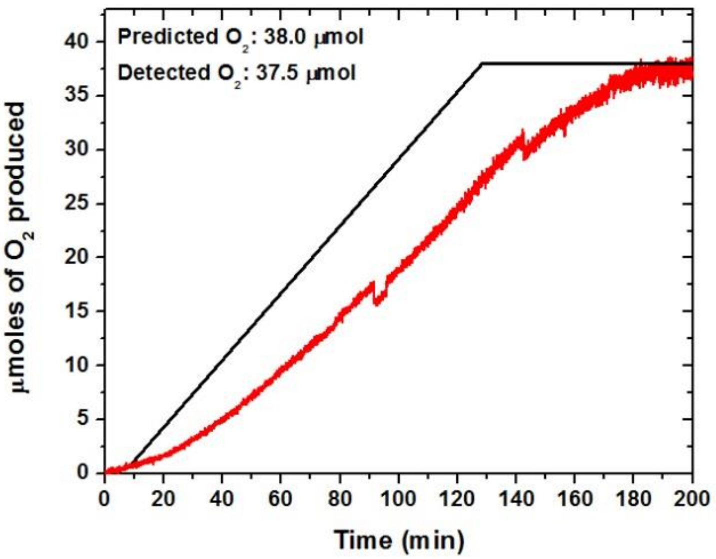

Supplementary Figure 23. Detected oxygen by phase fluorometry (red) and predicted maximum oxygen output from the electrode (black) calculated from current passed through the electrode. Lag time in detection is due to trapped oxygen bubbles that eventually escape to the headspace while stirring the electrolyte solution.

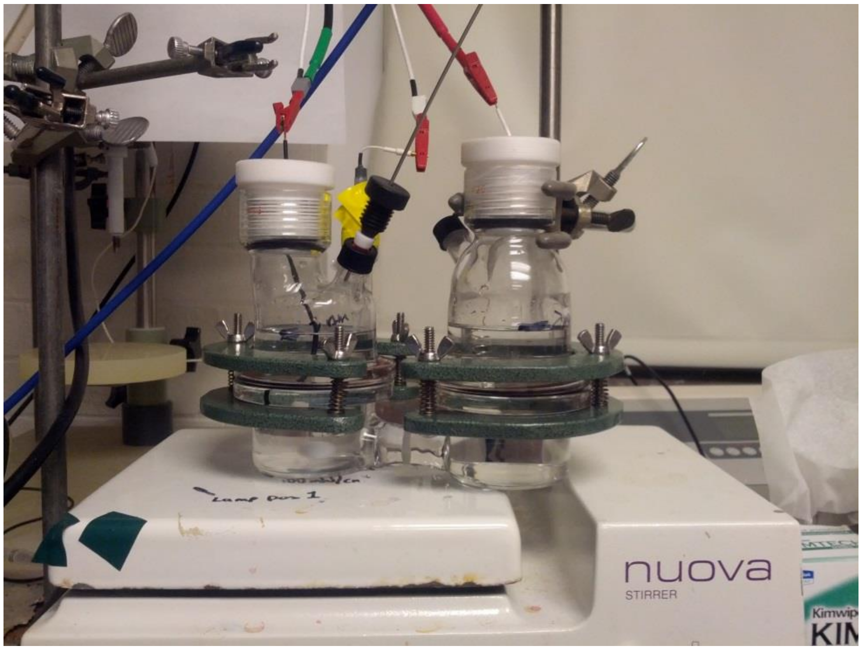

Supplementary Figure 24. Photograph of two-chamber oxygen detection set-up. Headspace volume noted in the experimental details above.

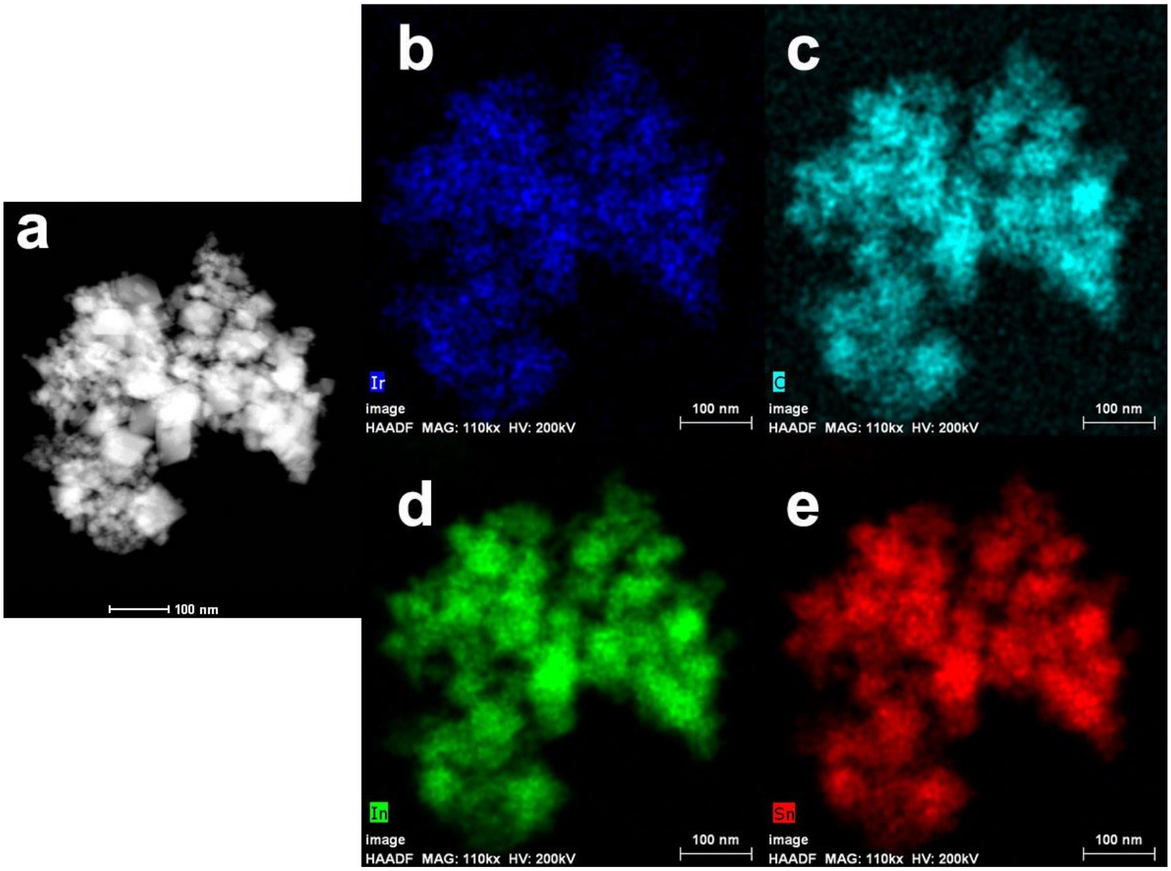

Supplementary Figure 25. HAADF (a) and STEM-EDX (b-e) maps taken using a post-electrolysis sample showing that Ir has not formed nanoparticles and still conformally coats the *nano*ITO particles. Color scheme follows Figure 7 in the manuscript. C is also shown (teal) in contrast to Supplementary Figure 26. However, sensitivity for C is poor in EDX because of both a low signal to noise ratio due to to adventitious carbon and the low atomic weight of C. XPS is therefore used to more accurately quantify carbon content, and the C XPS spectrum of an equivalent sample is shown in Figure 8 of the main text.

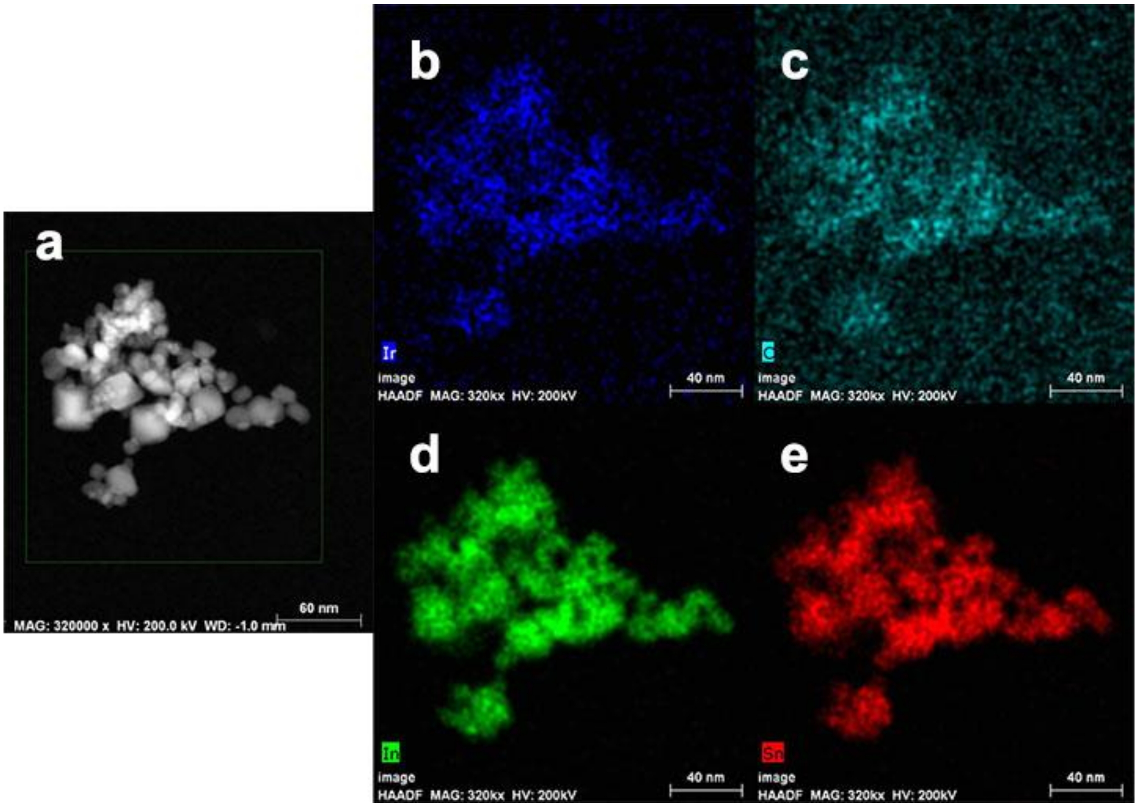

Supplementary Figure 26. Additional HAADF (a) and STEM-EDX (b-e) maps of a sample heated to 500 °C showing no nanoparticle formation. C is shown in teal and is significantly less than shown in an unheated sample, such as in Supplementary Figure 25. C XPS of an equivalent sample is shown in Supplementary Figure 27 (red trace).

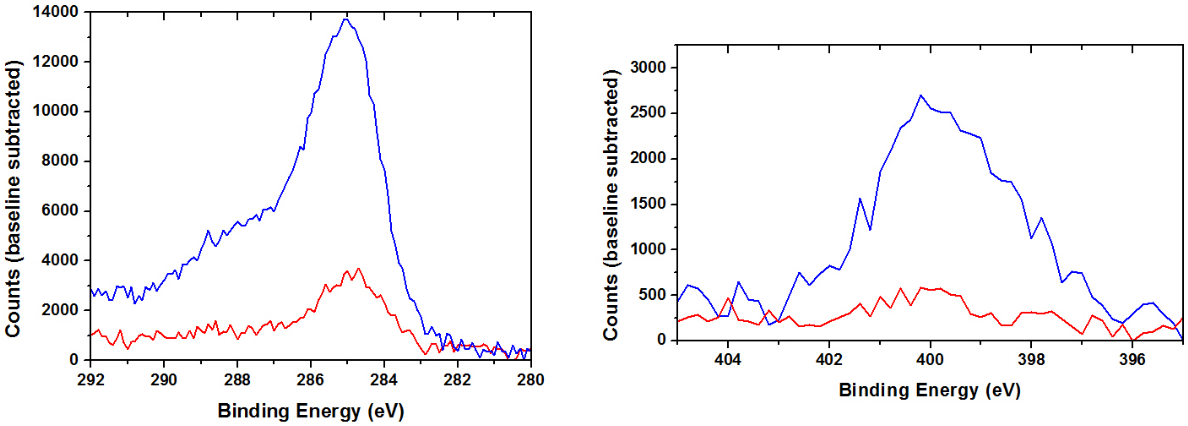

Supplementary Figure 27. XPS from an electrode heated to 500 °C (red) compared to one with catalyst as-deposited (blue, spectra from Figure 8 in the manuscript). Loss of C (left) aside from a small amount of adventitious C is clear, as well as loss of N (right). This corresponds to the loss of the pyalc ligand at 500 °C by oxidative removal in air.

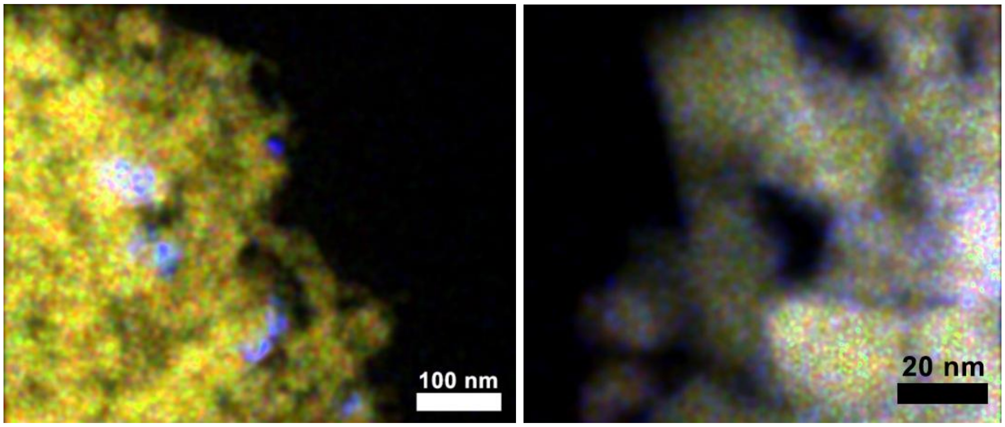

Supplementary Figure 28. A zoomed out STEM-EDX map of the nanoparticles formed by heating an electrode coated with Ir WOC to 700 °C is shown (left). All elements (Ir, Sn, In, using the same color scheme as in the main text) are detected, showing obvious iridium oxide nanoparticles formed in the *nano*ITO matrix. For comparison, the STEM-EDX maps shown in Figure 7 of an unheated sample are overlaid in a similar manner and displayed on the right.

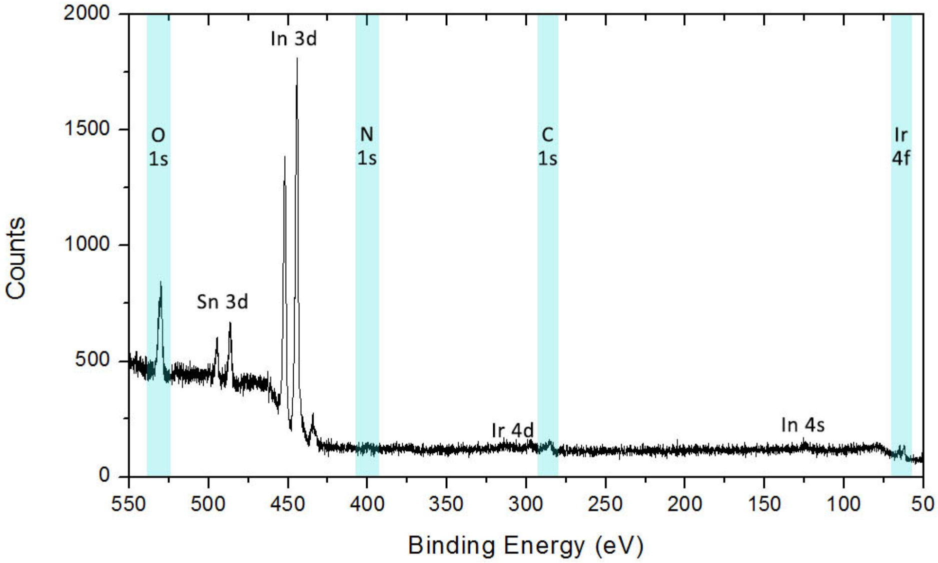

Supplementary Figure 29. XPS survey scan of the Ir WOC on *nano*ITO electrode, multiplexed high resolution scans are shown in Figure 8 of the main text. C, N, O, Ir, In, and Sn are the only elements detected.

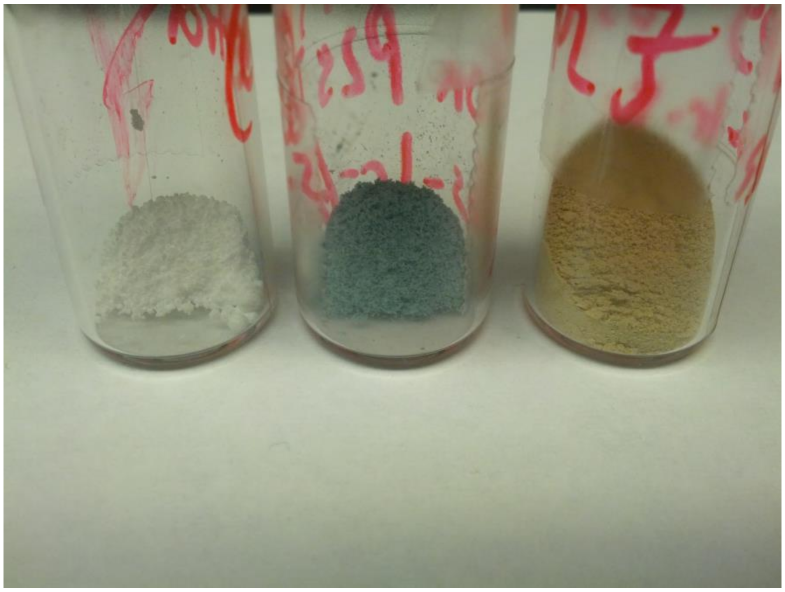

Supplementary Figure 30. Tunability of the catalyst's properties by changing ligands is optically evident. Left: bare TiO_2 powder, middle: TiO_2 powder with pyalc-bearing molecular Ir WOC bound to its surface, right: TiO_2 powder with bpy-bearing molecular Ir WOC bound to its surface.

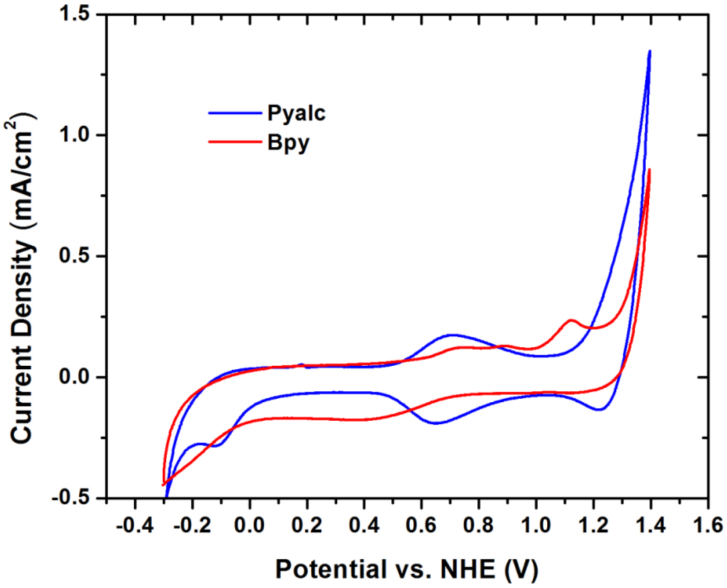

Supplementary Figure 31. CVs of *nano*ITO electrodes with Ir WOCs bearing two different bidentate chelating ligands. The bpy-bound Ir WOC possesses no similar features to the pyalc-bound one, and at no point during the CV did it turn blue. It also shows a new feature at 1.1 V vs NHE, reproducible in successive CVs.

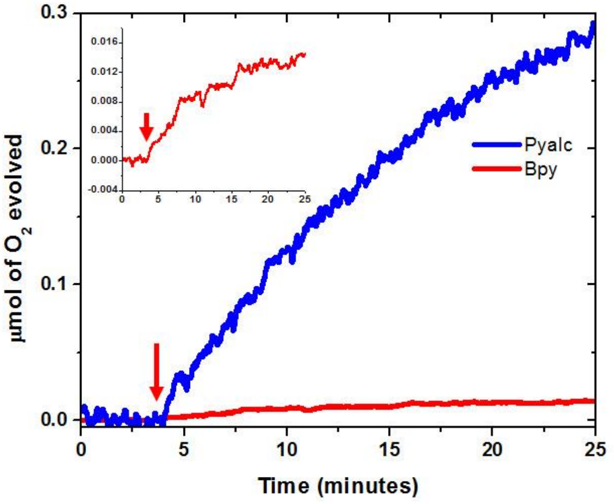

Supplementary Figure 32. Comparison of oxygen evolution activity for pyalc- and bpy-coordinated heterogeneous Ir WOCs using $NaIO_4$ as an oxidant under identical conditions (25 μL of 0.25 M $NaIO_4$ in deionized water, red arrows corresponds to injection). Inset shows the bpy-coordinated catalyst trace zoomed in, to show that oxygen is indeed detected, however, at a much lower rate. The same preparation procedures were used for both samples after formation of their respective homogeneous catalyst prior to heterogenization.

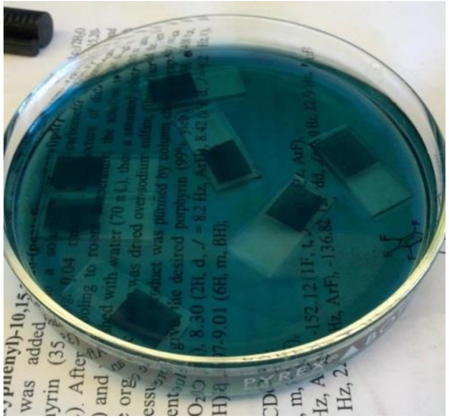

Supplementary Figure 33. Photograph of 1 cm^2 *nano*ITO on FTO-coated glass in a solution of the blue-colored WOC, $[Ir^{IV}(pyalc)(H_2O)_2(\mu\text{-}O)]_2^{2+}$ during heterogenization.

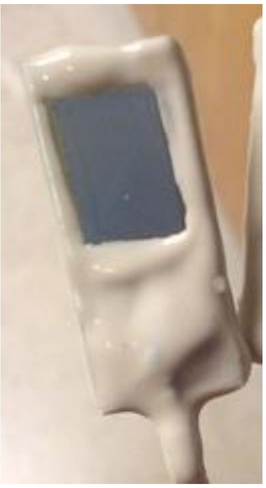

Supplementary Figure 34. Photograph of the type of electrode used for gathering Tafel plots and other electrochemical data.

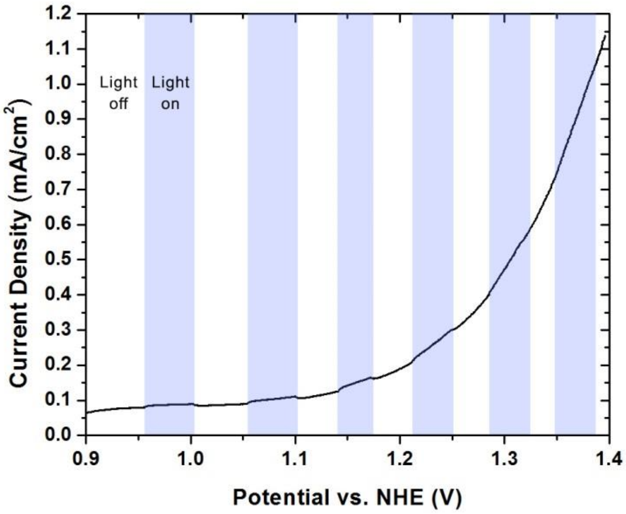

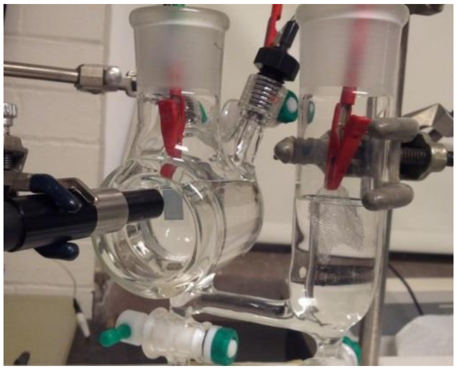

Supplementary Figure 35. Left: chopped light I-V scan to show no significant optical response from the catalyst on *nano*ITO, 50 mW/cm^2, 400 nm LP filtered Xe lamp, 10 mV/s scan rate. Right: Photograph of the experimental set-up.

Supplementary Tables

Peak	Position (eV)	Supplementary Refs.
Ir^{IV} 4f	7/2: 62.00, 5/2: 64.98	1-3
N 1s	399.76	4
C 1s (C-C)	284.93	4
C 1s (C-N-C)	286.60	4 and 5
C 1s (C-O)	288.56	4 and 6

Supplementary Table 1. Peak fit parameters for Figure 8, measurements taken with an Al anode (1486.6 eV), 35.75 eV pass energy, and Au as a calibration standard.

Supplementary Methods

Preparation of Working Electrodes. Working electrodes for electrochemical characterization were constructed by spin-coating at 1000 rpm on 2.2 mm thick glass slides coated with 500 nm of fluorine-doped tin oxide 30 seconds. *Nano*ITO was added to a mortar and pestle and ground for 10 minutes with glacial acetic acid, ethanol was then added to make a 5 M acetic acid/ethanol mixture, which was then sonicated for 10 minutes. A spin rate of 1000 rpm was used and amount of *nano*ITO in the 5 M acetic acid/ethanol mixture was varied between 5 wt% and 30 wt%, which resulted in films between approximately 300 nm and 7 µm. Greater thicknesses were achieved by successive spin coating of additional layers beyond the first, heating the particles on a hot plate to 200 °C for 10 minutes between coatings. For example, an 18 µm thick film was produced by spin coating three successive layers of 6 µm thick films using a 27 wt% *nano*ITO in 5 M acetic acid/ethanol solution. No boundaries between spin coated layers were observed in SEM for the thicker films. Heterogenization of the catalyst then follows the procedure described in the methods section of the manuscript, with a 30 minute wait time between mixing the precatalyst and $NaIO_4$ and immersion of the electrodes to ensure that no intermediate is being bound during its formation. Likely due to the oxidative stability of the pyalc ligand, we found no significant changes to the catalyst species or its electrochemistry when oxidizing the [Cp*Ir(pyalc)OH] precatalyst using $NaIO_4$ equivalents between 20 and 100.

TiO_2 slides were prepared by doctor blading a paste containing ~21 nm TiO_2 nanoparticles (P25, Sigma Aldrich) prepared according to published methods[7] on an FTO-coated glass slide, then heating in an oven to 450 °C in air for two hours. Heterogenization of $[Ir(pyalc)(H_2O)_2(\mu\text{-}O)]_2^{2+}$ on TiO_2 required only immersion of the substrate into the solution. A WO_3 paste for doctor blading onto an FTO-coated glass slide was made similarly to the *nano*ITO spin-coating paste, with 30 wt% WO_3 nanoparticles (<100 nm, Sigma Aldrich) in a 5 M acetic acid/ethanol mixture. After doctor blading onto an FTO-coated glass slide, the nanoparticles were heated in an oven to 550 °C in air for one hour. For attachment on WO_3, the pH of the homogeneous catalyst in solution was decreased to ~1.5 using a 1 M HNO_3 solution prior to immersion. The samples were allowed to sit for 12 hours (overnight) to ensure complete binding, then they were removed and washed thoroughly with deionized water.

Effect of solution pH and iodate concentration on surface binding experiments. These were performed on catalyst deposited on 3.5 µm thick spin-coated P25 TiO_2 on glass cover slips because of the higher stability of TiO_2 at varied pH conditions when compared with ITO, which allowed for more accurate characterization. For each experiment, slides of spin-coated TiO_2 were immersed in catalyst solutions, removed after 1 hour, washed thoroughly with deionized water, and measured using an integrating sphere. These results were all reproduced in triplicate for accuracy. The trends present in the amount of catalyst bound to the surface, as measured by the catalyst's absorption of visible light, are then used to compare the effect of solution conditions on rate of surface binding between samples.

It is important to note that all samples contain a small amount of iodate anion and degradation products from Cp* due to its oxidative removal from the precatalyst using $NaIO_4$ (which decays to $NaIO_3$) for initial formation of the active catalyst, however the concentration is significantly smaller than the amounts of added iodate in Supplementary Figure 9. The native pH of the solution upon oxidation of precatalysts with $NaIO_4$ is slightly acidic (4-5), at which point there is

a 0.5 ratio of aqua and hydroxo bound Ir centers.[8] The data in Supplementary Figure 9 shows that increasing the amount of aqua bound Ir by lowering the homogeneous catalyst solution pH greatly increases the rate of catalyst adhesion to the surface; while increasing the solution pH, which deprotonates the aqua ligands thereby forming hydroxo-bound Ir centers, causes surface-binding to be inhibited.

Electron Paramagnetic Resonance (EPR). EPR spectroscopy was performed on a Bruker ELEXSYS E500 EPR spectrometer equipped with a SHQ resonator and an Oxford ESR-900 helium-flow cryostat. A microwave frequency of 9.4 GHz with 10 G modulation amplitude was used, at a microwave power of 1 mW. The temperature in the cryostat was held constant between 7.5 K and 8 K using liquid helium. *Nano*ITO used as a substrate for the surface-bound catalyst due to its high surface area which allowed for a sufficient overall amount of catalyst, significantly greater than the detection limit of the instrument, to be loaded into capillary tubes for analysis. The catalyst-bound nanoparticles were made into a thick slurry with water and packed into 3 10 μL capillary tubes, which were then sealed with a clay sealing compound (corresponding to some of the background features in the spectra) and loaded into an EPR tube. Acetone was added to the EPR tube to surround the capillaries and provide thermal conductivity with the cryostat, and the tube was degassed using dry N_2 to remove atmospheric oxygen.

We can compare this to the EPR spectrum of the **hom-WOC**[8] to see that in both cases the Ir^{IV} dimer species is not EPR-active. If a monomeric Ir^{IV} species were present on the surface, we would expect it to be EPR-active and have a spectrum similar to what was observed in Brewster *et al*.[9] Due to the lack of Ir –related features in the EPR spectra and no discernable signal difference spectrum shown above, we can therefore postulate that the Ir^{IV} compound that we previously demonstrated to be a dimer in solution remains in dimer form in its resting Ir^{IV} state when bound to the surface of metal oxides. To control for any interactions with the conductive *nano*ITO the experiments were reproduced on P25 TiO_2, which is a much better insulator, and no EPR-active Ir^{IV} was found. Powers up to 20 mW were also tested, and no EPR-active Ir^{IV} was found.

Water Oxidation using $NaIO_4$. Control experiments included *nano*ITO on FTO-coated glass samples without any catalyst added, and that were soaked in the precatalyst solution. No catalytic activity was found in any of the control experiments, as shown in Supplementary Figures 3 and 4.

In Figure 3a, a sample was used that was loaded with approximately 49.1 nmol of iridium. Decreasing the amount of catalyst on the surface by decreasing *nano*ITO thickness, we were able to achieve higher turnover numbers and turnover frequencies as seen in Supplementary Figure 15 due to both a larger ratio of oxidant to catalyst and a higher percentage of the catalyst easily accessible to the solution without having to diffuse oxidant through the mesoporous *nano*ITO film.

Determination of catalyst loading on electrodes. A brief description of this procedure is included in the methods section of the manuscript. Specifically, when preparing heterogeneous catalyst samples of Ir WOC bearing pyalc ligands, the absorption of the homogeneous catalyst solution at 580 nm was monitored after introduction of a substrate, with a control lacking *nano*ITO as a baseline. Fresh glassware must be used for each measurement due to the catalyst's ability to bind to numerous metal oxides, including SiO_2. For electrochemical measurements

mentioned in the text, electroactive iridium was used to gauge catalytic activity instead since it could be determined with higher accuracy using CV peak integration of the Ir^{III}/Ir^{IV} redox feature. Using both of these measurements, the ratio of electroactive Ir to Ir present on the surface detected by loss in absorption of the homogeneous catalyst solution during preparation was typically >90% (± 5%). When not in a gas-tight cell performing phase fluorometric oxygen detection, turnover numbers (TON) were estimated by the current passed through the electrode in constant-current chronopotentiometry experiments, assuming a four-electron process for water oxidation:

$$\boldsymbol{TON = \frac{It}{4FN_{Ir}}} \qquad (1)$$

Where I is the current passed through the electrode in amperes, t is the time over which the current was passed in seconds, F is the Faraday constant (96485 C/mol), and N_{Ir} is the amount of electroactive iridium measured by the aforementioned CV peak integration in moles. Electrochemical measurements were performed using a Princeton Applied Research Versastat 4-400 in a standard three electrode configuration, CVs were taken with a 5 second equilibration time at their starting potential prior to data collection.

Oxygen detection and faradaic yield. Headspace oxygen detection was performed using a TauTheta MFPF-100kHz phase fluorometric oxygen detection system with a FOSPOR-R probe (Ocean Optics). The experiment was performed in a custom-built two chamber gas-tight electrochemical cell (Suppementary Figure 23). A sample made for long-term electrolysis was constructed using conductive epoxy to secure a wire to bare FTO on a 6.45 cm^2 geometric surface area FTO-coated glass slide covered with a *nano*ITO film <300 nm thick. The conductive epoxy was then coated with white marine epoxy, an example of this electrode design is shown in Supplementary Figure 20. The two-chamber cell was fitted with an Ag/AgCl reference electrode in the working electrode chamber and a Pt electrode in the counter electrode chamber an filled with pH 2.6, 0.1 M KNO_3 in deionized water. The FOSPOR-R probe was inserted into a rubber septum that was secured on one of the apertures in the working electrode chamber of the electrochemical cell, and all connections lacking an O-ring were wrapped tightly with Parafilm and electrical tape. Both chambers were degassed under vigorous stirring with high purity N_2 for over 2 hours using needles inserted into rubber septa bubbling through the electrolyte solution, while monitoring oxygen content to ensure that O_2 in the system was minimal. The needles were then removed and the purge was stopped, and O_2 levels were monitored for 30 minutes with no increase in O_2 concentration to ensure a stable, oxygen-free atmosphere had been achieved.

A constant overpotential of 520 mV was applied for two hours and the results are shown in Supplementary Figure 23. Bubble formation on the working and counter electrodes was immediately visible upon applying the potential. Vigorous stirring was necessary in order to prevent bubble accumulation on the surface of the working electrode. This did cause some oxygen bubbles to become trapped near the glass frit or the O-ring holding the working electrode chamber together, causing a lag time between O_2 bubble generation and detection in the headspace. The volume of gas in the headspace of the working electrode chamber was measured to be 38 mL. CVs were taken both before and after electrolysis with little change, showing minimal loss of catalyst over the course of the experiment. By integration of the Ir^{III}/Ir^{IV} redox

wave, total electroactive iridium was determined to be ~0.66 nmol. This corresponds to approximately 7.9 (± 0.6) turnovers of O_2 per second per iridium atom, and a turnover number of 56,800 calculated by dividing the total number of moles of oxygen detected by the time an electric potential was applied to the electrode, then dividing that number by the number of moles of electroactive iridium atoms present. Faradaic yield was calculated to be 98.7%. No significant current or oxygen generation was found at the same potential from a similar *nano*ITO on FTO-coated glass electrode without the pyalc Ir WOC on the surface as a control.

Additional stability data. We found that the stability of the electrodes was highly reproducible given the correct conditions, including either a buffered solution at low potentials, or an unbuffered solution using a thin *nano*ITO film and the electrolyte stirred vigorously to prevent the buildup of a pH gradient that leads to *nano*ITO etching. During TEM and SEM analysis, we examined electrodes both before and after long-term electrolysis to show that there is no change to sample morphology or nanoparticle formation. Supplementary Figure 21 shows one of these electrodes that underwent hours of catalytic water oxidation at approximately 250 mV overpotential with little to no observed decline in activity.

We also tested the stability of our catalyst on the surface at higher applied potentials. At applied potentials as high as 2 V vs. NHE (approx. 2.15 V vs RHE), we see no catalyst degradation and high current densities limited primarily by mass transport due to rapid bubble formation (Supplementary Figure 22).

Tafel plots, pH dependence, and KIE. Tafel plots shown in Figure 5 were gathered using a Pt mesh counter and Ag/AgCl reference electrode while the solution was stirred, with standard electrolyte conditions except where noted in the manuscript. 25 mV steps with a 5 second rest time between steps were used, beginning at 0.750 V vs Ag/AgCl and ending at 1.400 V vs Ag/AgCl. No detectable current above the level of noise intrinsic to the experiment was found at applied potentials below the thermodynamic potential for water oxidation. While electrodes reached a stable current density in less than 1 minute at each step, experiments used up to 5 minutes of chronoamperometry per step to ensure that electrodes were adquately stabilized at each point. Freshly prepared electrodes and electrolyte solutions were always used for Tafel plots.

Electrodes prepared for this and other electrochemical measurements such as CVs, pH dependence, and thickness dependence used a geometric active area of 1 cm^2. A wire was attached to the FTO substrate using conductive epoxy, and the entire electrode aside from the active area was encased in non-conductive marine epoxy to ensure no contribution to current from catalyst bound to FTO or glass. A photograph of this type of electrode is shown in Supplementary Figure 34.

Data for pH dependence (Supplementary Figure 19) were taken using 7 μm thick *nano*ITO films on FTO-coated glass with 0.1 M KNO_3 as the electrolyte. The data both were taken using chronopotentiometry and extrapolated from Tafel plots taken at different pHs for accuracy and determination of error. At low and high pHs, H^+ and OH^- behave as buffers causing a smaller rate of change for overpotential versus pH than at neutral pHs. This effect was seen in previous studies with BL, and is due to the low buffering capacity of KNO_3 at these pHs. The pH

dependence can be changed by addition of a compound with higher buffering capacity at these pHs, as shown in Figure 5.

A preliminary measurement of H_2O/D_2O kinetic isotope effect (KIE) was taken under standard electrolyte conditions and is shown in Supplmentary Figure 17, with the pH and pD of the solution made equivalent according to previously published methods.[10] We found a KIE of approximately 1 at low applied overpotentials, similar to IrO_2 in photodriven schemes.

Light on/off control experiments and other electrochemical controls. Due to the low overpotential of this catalyst and its strong absorption at 580 nm, it is reasonable to have the suspicion that some of the energy required to split water may come from ambient light or some other external energy source. We demonstrate that this is not the case by performing experiments both in light-on and light-off conditions (Supplementary Figure 35). To more rigorously examine this, we performed experiments with a Xe lamp and 400 nm longpass filter. The results of an IV-curve under chopped illumination are shown, taking using a two-chamber photoelectrochemical cell (all electrochemical conditions otherwise standard).

Additional controls. Additional controls include CVs of *nano*ITO electrodes soaked for up to 48 hours in non-activated [Cp*Ir(pyalc)OH] precatalyst solutions or in a solution of free pyalc ligand, removed from those solutions and washed, then placed into an electrochemical cell. Controls were also performed with electrodes soaked in $NaIO_3$ or $NaIO_4$ without catalyst for 48 hours, and electrodes heated to 500° C and 700° C prior to catalyst deposition; no significant difference was found aside from an increase in electrode resistance due to the lower conductivity of *nano*ITO in the heated electrodes. Further control experiments were performed by combining the [Cp*Ir(pyalc)OH] with $NaIO_3$ in deionized water at the same concentrations as the **hom-WOC** (the major difference being the presence of the Cp* ligand that would be removed if $NaIO_4$ was used instead) and immersing a *nano*ITO on FTO-coated glass electrode in to the solution for 48 hours. We observed no deposition of catalyst in this case as well, demonstrating that Cp* removal is required to open the coordination sites needed for surface binding. In all cases, CVs of the electrodes after having been removed from the [Cp*Ir(pyalc)OH]-containing solution and washed with deionized water looked identical to bare *nano*ITO electrodes.

SEM/TEM additional experimental details. Both silicon monoxide and holey carbon grids were used in this study. Using an SiO grid allowed us to monitor carbon content with moderate accuracy due to noise from adventitious carbon; much more accurate analysis of carbon present was done by XPS. TEM images as well as STEM-EDX maps were taken on different samples using the same conditions for all samples, both before and after electrolysis, and after heating to 500 °C and 700 °C.

Additional XPS details. Supplementary Figure 29 shows a survey scan of the electrode. Multiplexed scans from the same electrode are shown in Figure 8 of the main text. XPS analysis showed an Ir 4f doublet at 62.00 and 62.98 eV, values that have been seen previously with iridium oxide materials that possess Ir in the IV oxidation state.[1] Our results are also fully consistent with Ir being in the IV oxidation state when compared to other molecular Ir^{IV} compounds,[3] and are similar to what we saw previously with the catalyst in a homogeneous environment.[8] As a further comparison, the Ir 4f doublet for this catalyst is shifted to a

considerably higher binding energy than previously reported Ir^{III} compounds.[2] The N 1s signal at 399.76 eV is consistent with literature for pyridyl N coordinated to a metal,[5] as well as the C 1s (C-N-C)[5,6] and C 1s (C-O)[6] peaks. The C 1s (C-C) peak is inclusive of adventitious carbon;[4] however, its intensity is higher than we would expect if that were the sole source of C in that peak suggesting contribution from the pyalc ligand.

XPS of pyalc ligand loss from an electrode heated to 500 °C. Upon heating to 500 °C in air, the N 1s, C 1s (C-O), and C 1s (C-N-C) signals disappear, while a much smaller amount of adventitious carbon at 284.93 eV remains (Supplementary Figure 27). This provides evidence that the pyalc ligand is removed upon heating an electrode with Ir WOC heterogenized on the surface to this temperature.

XPS Data shown in Supplementary Figure 10. Data was collected using a commercial Thermo Scientific K-Alpha^{+} XPS system with a dual-beam flood ion source illuminating the sample for charge compensation during analysis. An Al Kα x-ray monochromator (hν = 1486.6 eV) with a 400 μm spot size was used. The spectra were referenced using adventitious carbon (284.8 eV).

Ligand Tunability: Preliminary data with $[Ir(bpy)(H_2O)_2(\mu\text{-}O)]_2^{n+}$. The precatalyst $[Cp^*Ir(bpy)OH]BF_4$ and the catalyst formed by oxidation of that compound, previously proposed to be $[Ir(bpy)(H_2O)_2(\mu\text{-}O)]_2^{n+}$, was synthesized according to our prior published methods using 20 equivalents of $NaIO_4$.[8] A dependence on $NaIO_4$ equivalents added was observed in our experiments for catalysts bearing a bpy ligand. Such a dependence has been recently explored in homogeneous systems using bpy-based catalysts by Lewandowska-Andralojc *et al*,[11] and we plan to explore this further in the future in our heterogeneous systems. Heterogenization onto *nano*ITO, electrode construction, and all other parameters not outlined here were the same as the experiments performed with the pyalc ligated catalyst.

The most striking difference is the obvious disparity in color between the surface-bound Ir WOC bearing a pyalc or bpy ligand. To show this color change based on the ligand, we deposited the two catalysts on white, opaque P25 TiO_2 (Supplementary Figure 30). One hypothesis as to the cause of this difference in color for the molecular heterogeneous catalysts is that the anionic N-O pyalc ligand may stabilize the blue Ir^{IV} state, while it may not be stabilized with a neutral N-N ligand such as bpy.

Looking at the CVs (Supplementary Figure 31), the bpy-bound Ir WOC does not have a well-defined Ir^{III}/Ir^{IV} redox wave as well, but does possess a feature at 1.1 V vs NHE that is not correlated with a change in color of the electrode. Our further studies will explore these electrochemical differences further; however, it is possible that a less stable Ir^{V} state due to the pyalc ligand could account for the higher activity toward water oxidation that the pyalc-bound Ir WOC possesses in both homogeneous and heterogeneous catalytic environments.

Supplementary References

1. Rubel, M. *et al.* Characterization of IrO_2-SnO_2 Thin-Layers by Electron and Ion Spectroscopies. *Vacuum* **45**, 423-427 (1994).

2. Wang, C., Wang, J. L. & Lin, W. B. Elucidating Molecular Iridium Water Oxidation Catalysts Using Metal-Organic Frameworks: A Comprehensive Structural, Catalytic, Spectroscopic, and Kinetic Study. *J. Am. Chem. Soc.* **134**, 19895-19908 (2012).

3. Kim, Y. I. & Hatfield, W. E. Electrical, Magnetic and Spectroscopic Properties of Tetrathiafulvalene Charge-Transfer Compounds with Iron, Ruthenium, Rhodium and Iridium Halides. *Inorg. Chim. Acta* **188**, 15-24 (1991).

4. Barr, T. L. & Seal, S. Nature of the use of adventitious carbon as a binding energy standard. *J. Vac. Sci. Technol., A* **13**, 1239-1246 (1995).

5. Xue, G., Dai, Q., Jiang, S. Chemical Reactions of Imidazole with Metallic Silver Studied by the Use of SERS and XPS Techniques. *J. Am. Chem. Soc.* **110**, 2393-2395 (1988).

6. Weldon, M. K., Uvdal, P., Friend, C. M. & Serafin, J. G. Decoupling of vibrational modes as a structural tool: Coverage-induced reorientation of methoxide on Mo(110). *J. Chem. Phys.* **103**, 5075-5084 (1995).

7. Ito, S. *et al*. Fabrication of Screen-Printing Pastes From TiO_2 Powders for Dye-Sensitized Solar Cells. *Prog. Photovolt: Res. Appl.* **15**, 603-612 (2007).

8. Hintermair, U. *et al.* Precursor Transformation during Molecular Oxidation Catalysis with Organometallic Iridium Complexes. *J. Am. Chem. Soc.* **135**, 10837-10851 (2013).

9. Brewster, T. P. *et al.* An Iridium(IV) Species, $[Cp^*Ir(NHC)Cl]^+$, Related to a Water-Oxidation Catalyst. *Organometallics* **30**, 965-973 (2011).

10. Glasoe, P. K. & Long, F. A. Use of Glass Electrodes to Measure Acidities in Deuterium Oxide. *J. Phys. Chem.* **64**, 188-190 (1960).

11. Lewandowska-Andralojc, A. *et al.* Efficient water oxidation with organometallic iridium complexes as precatalysts. *Phys. Chem. Chem. Phys.* **16**, 11976-11987 (2014).
